# Supplementary material for: Long-read assembly of the Chinese rhesus macaque genome and identification of ape-specific structural variants
Source: Nat Commun. 2019 Sep 17;10:4233. doi: 10.1038/s41467-019-12174-w (PMC6749001; doi:10.1038/s41467-019-12174-w)
Supplement: Supplementary file 1 — Supplementary Information [file 41467_2019_12174_MOESM1_ESM.pdf]

Supplementary Information for

Long-read assembly of the Chinese rhesus macaque genome and identification of ape-specific structural variants

Yaoxi He*, Xin Luo*, Bin Zhou*, Ting Hu*, Xiaoyu Meng*, Peter A. Audano, Zev N. Kronenberg, Evan E. Eichler, Jie Jin, Yongbo Guo, Yanan Yang, Xuebin Qi, Bing Su†

*These authors contributed equally to this work.
†Corresponding author. E-mail: sub@mail.kiz.ac.cn

This PDF file includes:
Supplementary Figures 1-28
Supplementary Tables 1-22
Supplementary Data are provided as separate Excel files.

Supplementary Figures

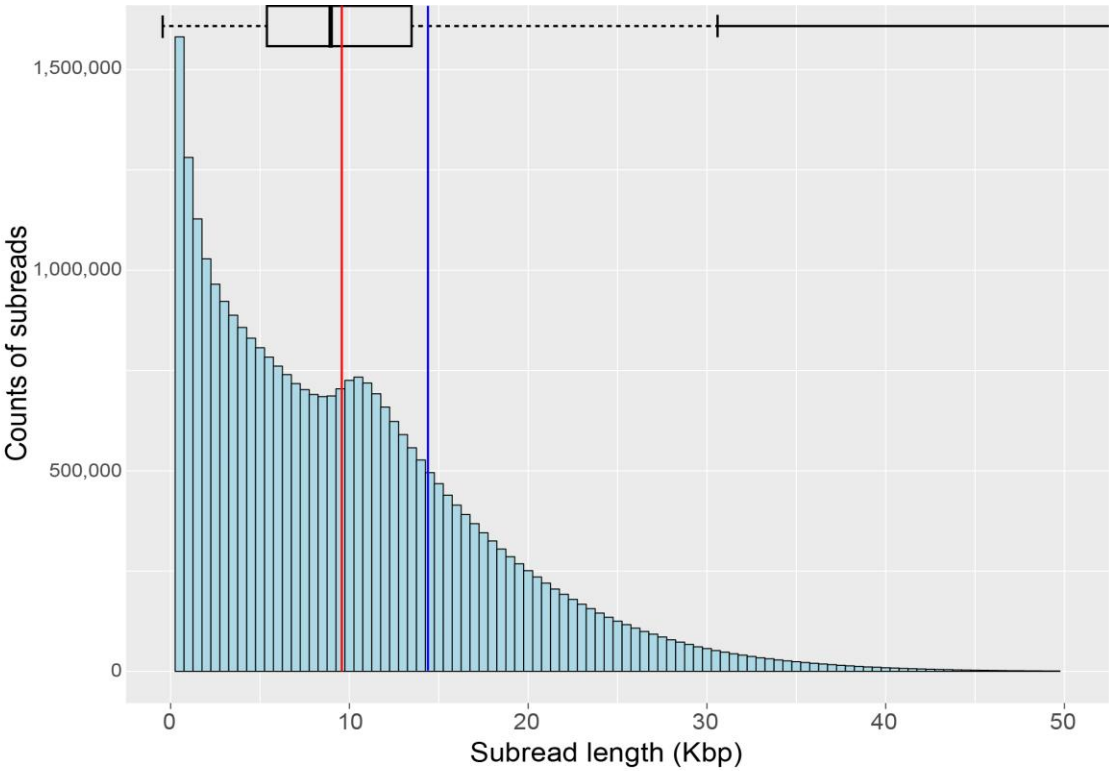

Supplementary Figure 1. Distribution of subread length of rheMacS. Marginal box plot indicates quartiles. The mean subread length is 9.7 Kbp (red line) and the N50 subread length is 14.7 Kbp (blue line).

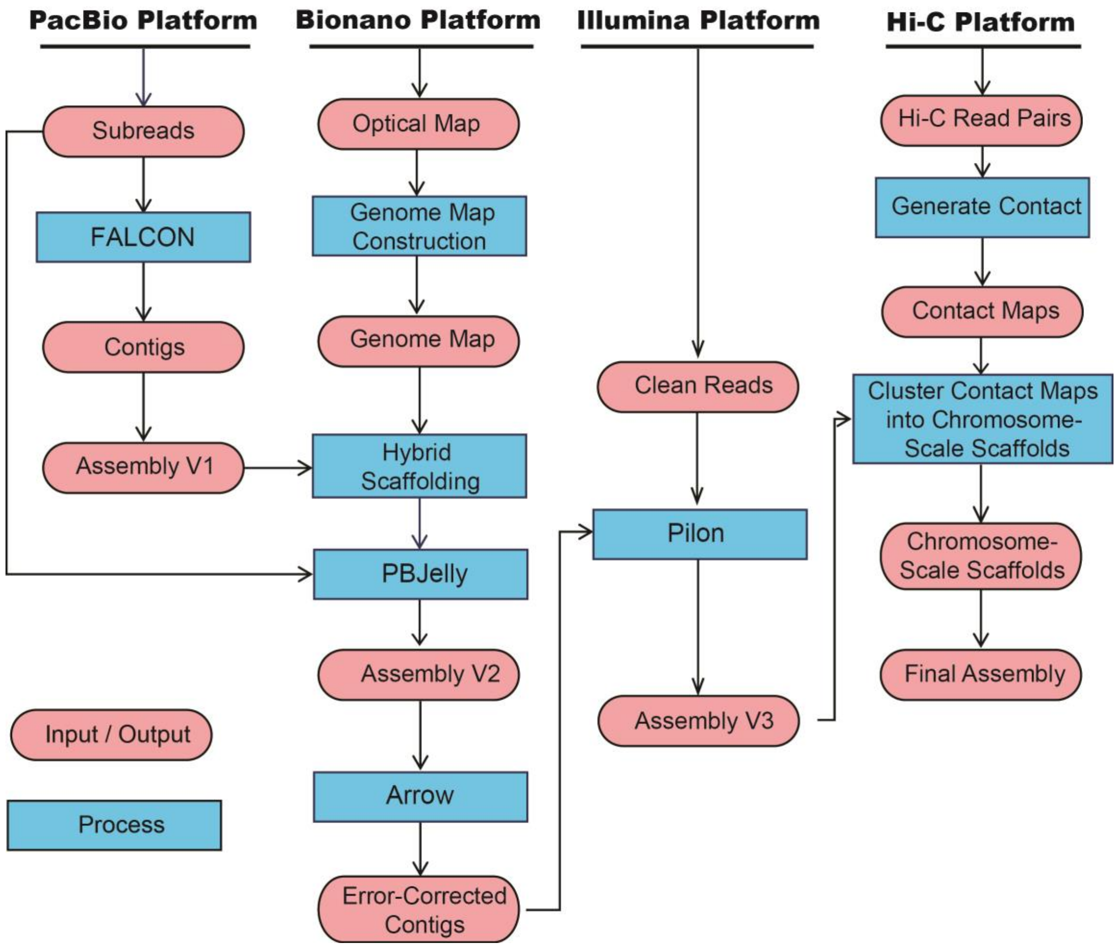

Supplementary Figure 2. Data generation and *de novo* assembly pipeline.

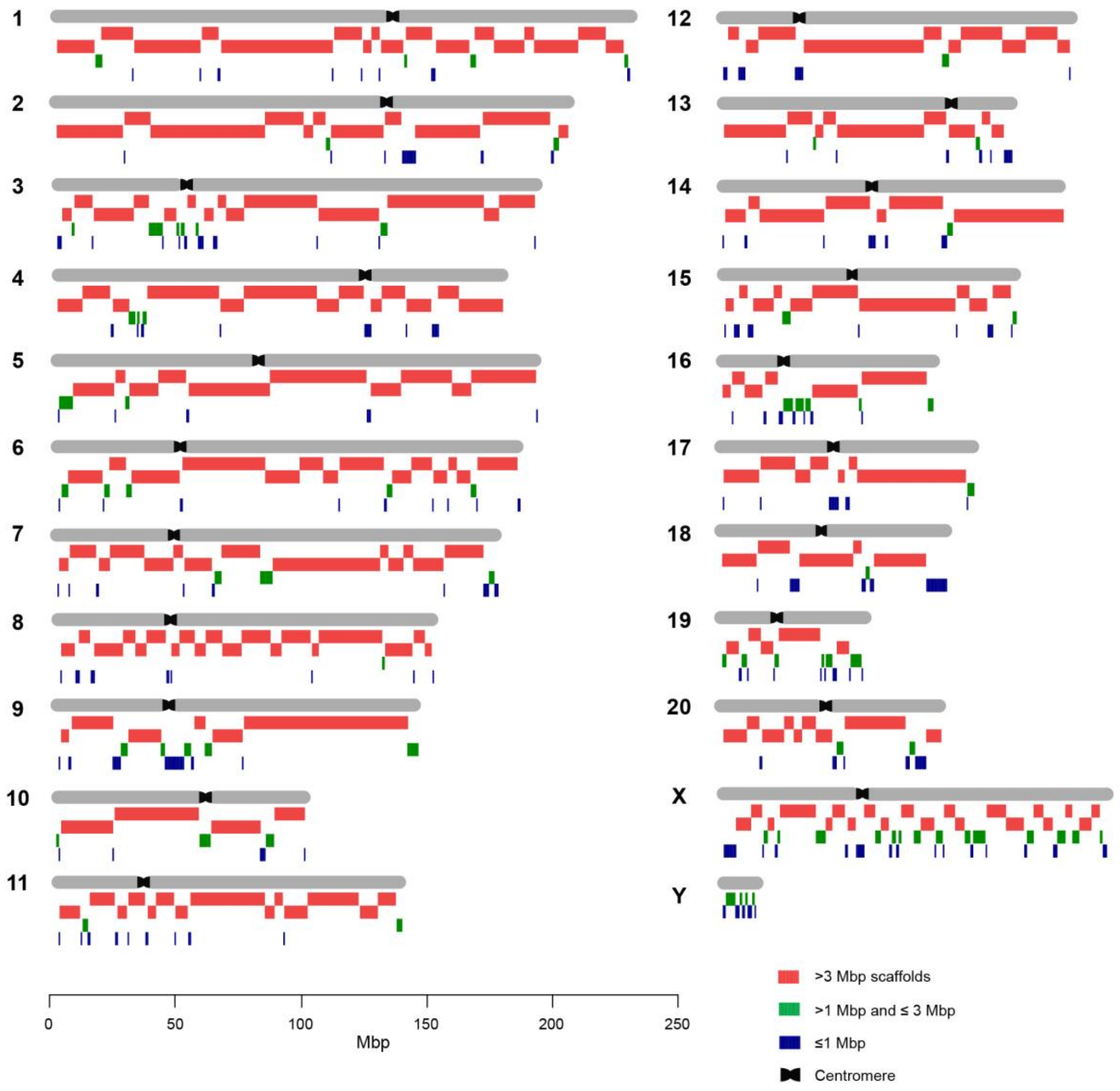

Supplementary Figure 3. The chromosomal distribution of scaffolds of the rheMacS genome assembly. The red rectangles represent scaffolds >3 Mbp; green and blue rectangles correspond to the scaffolds with lengths between 1 Mbp and 3 Mbp and those <1 Mbp, respectively.

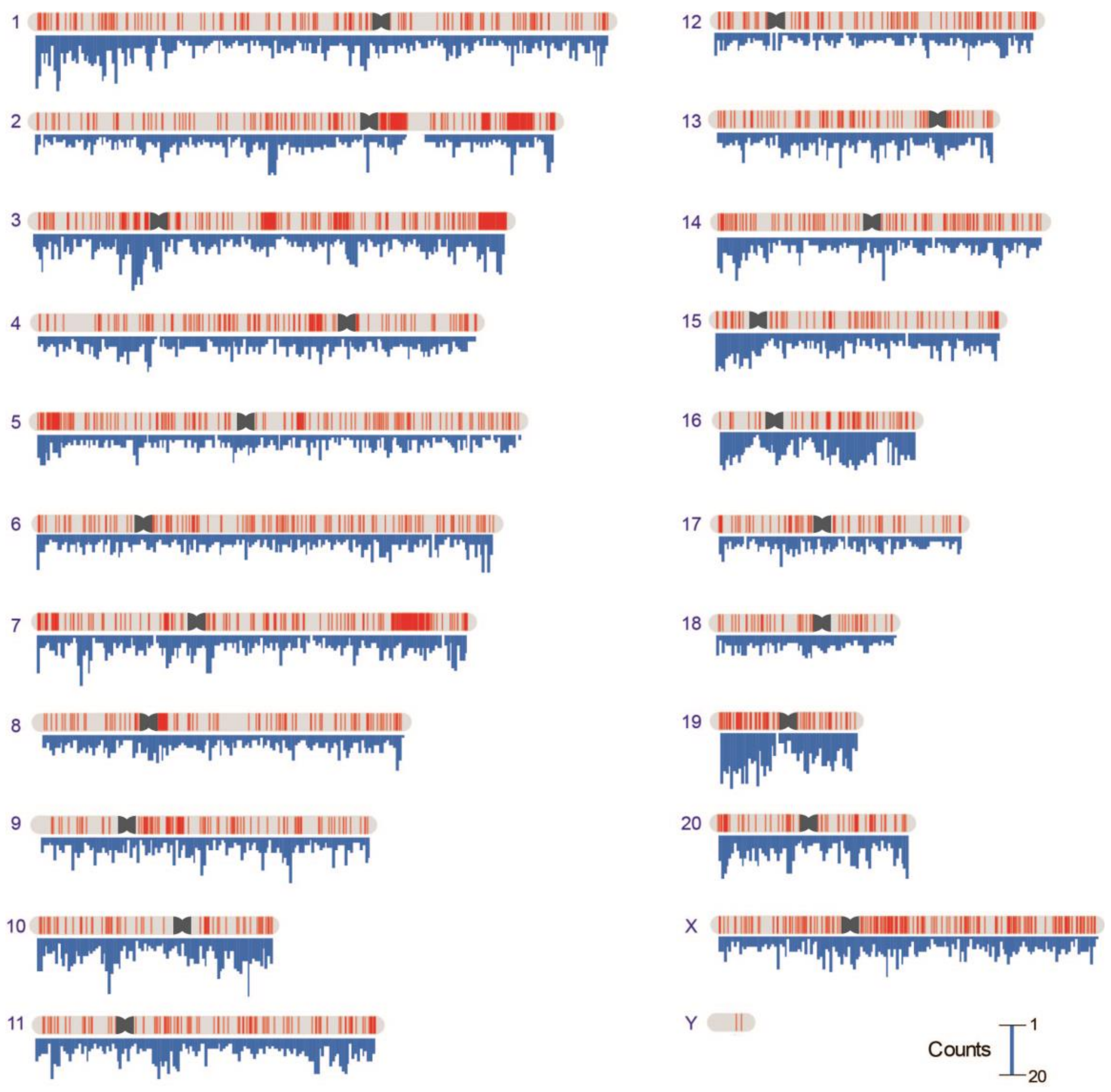

Supplementary Figure 4. Distribution of closed gaps in rheMac8 (only the >1 Kbp closed gaps are shown). The blue-colored histograms on the axes of each chromosome indicate the counts of the closed gaps based on 500 Kbp windows. The black dot on each chromosome indicates the centromere position.

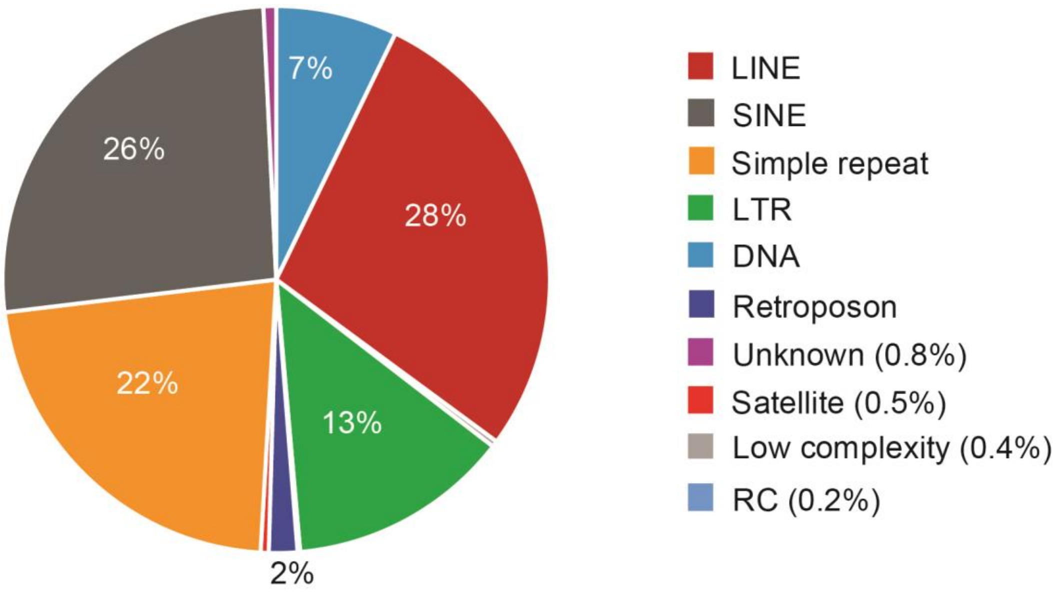

Supplementary Figure 5. Repeat annotation for the closed gaps in rheMac8.
LINE: long interspersed nuclear elements; SINE: short interspersed nuclear elements; LTR: long terminal repeats; RC: rolling-circle transposition.

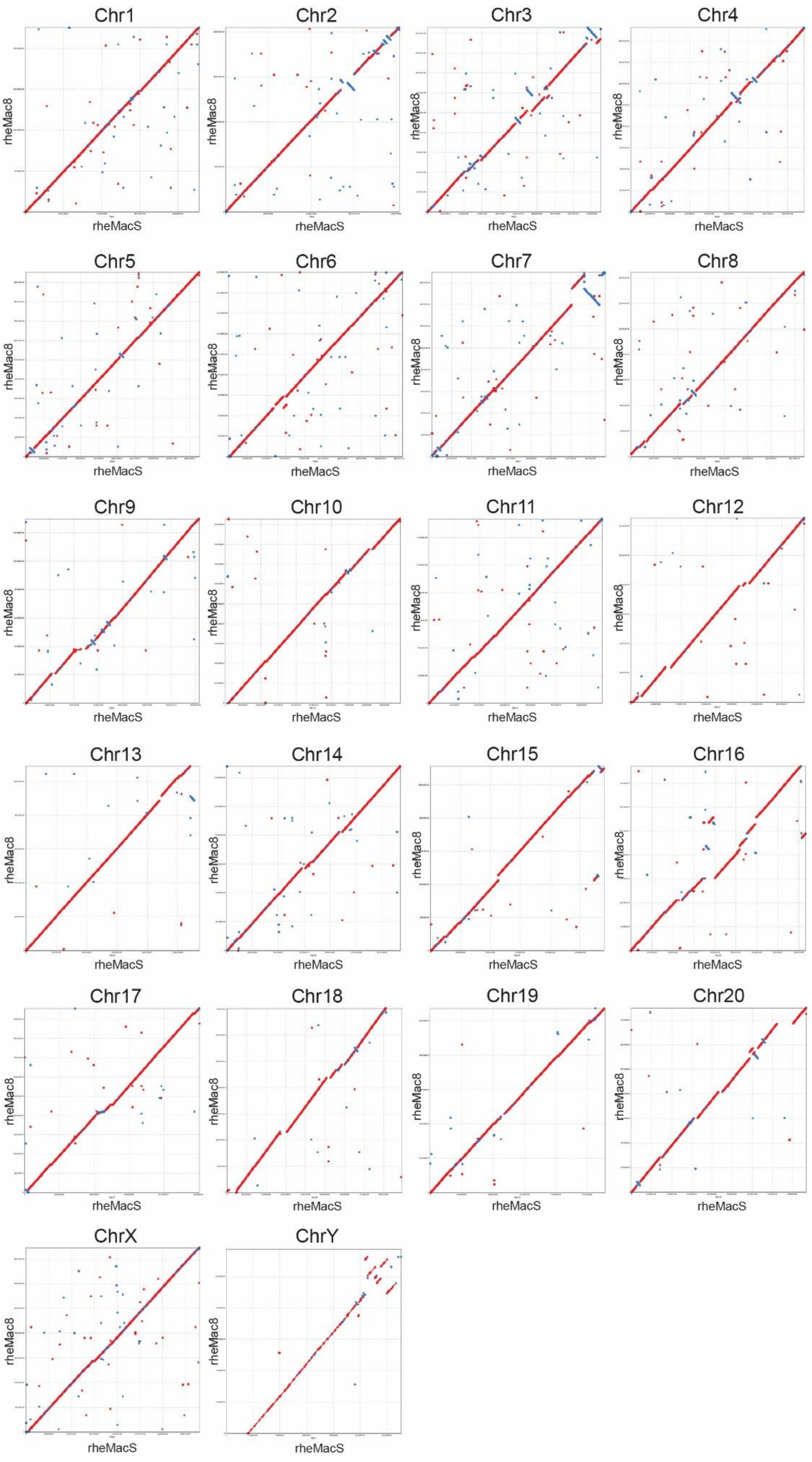

Supplementary Figure 6. Dot plots of assembly comparison between rheMacS and rheMac8.

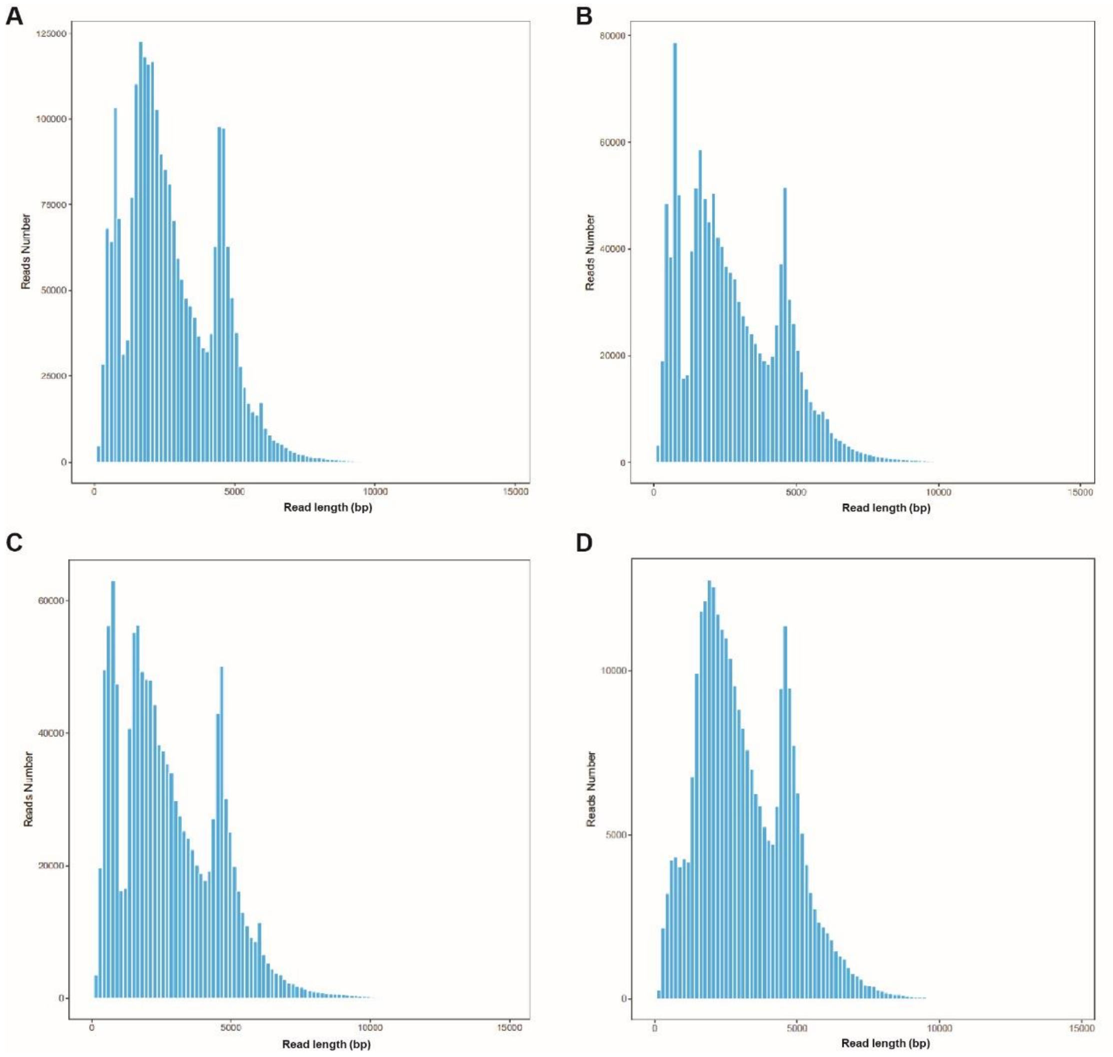

Supplementary Figure 7. Distribution of transcripts. (A) Length distribution of Full-length non-chimeric (FLNC) reads. **(B)** Length distribution of consensus sequences. **(C)** Length distribution of the NGS-corrected reads. **(D)** Length distribution of collapsed isoforms.

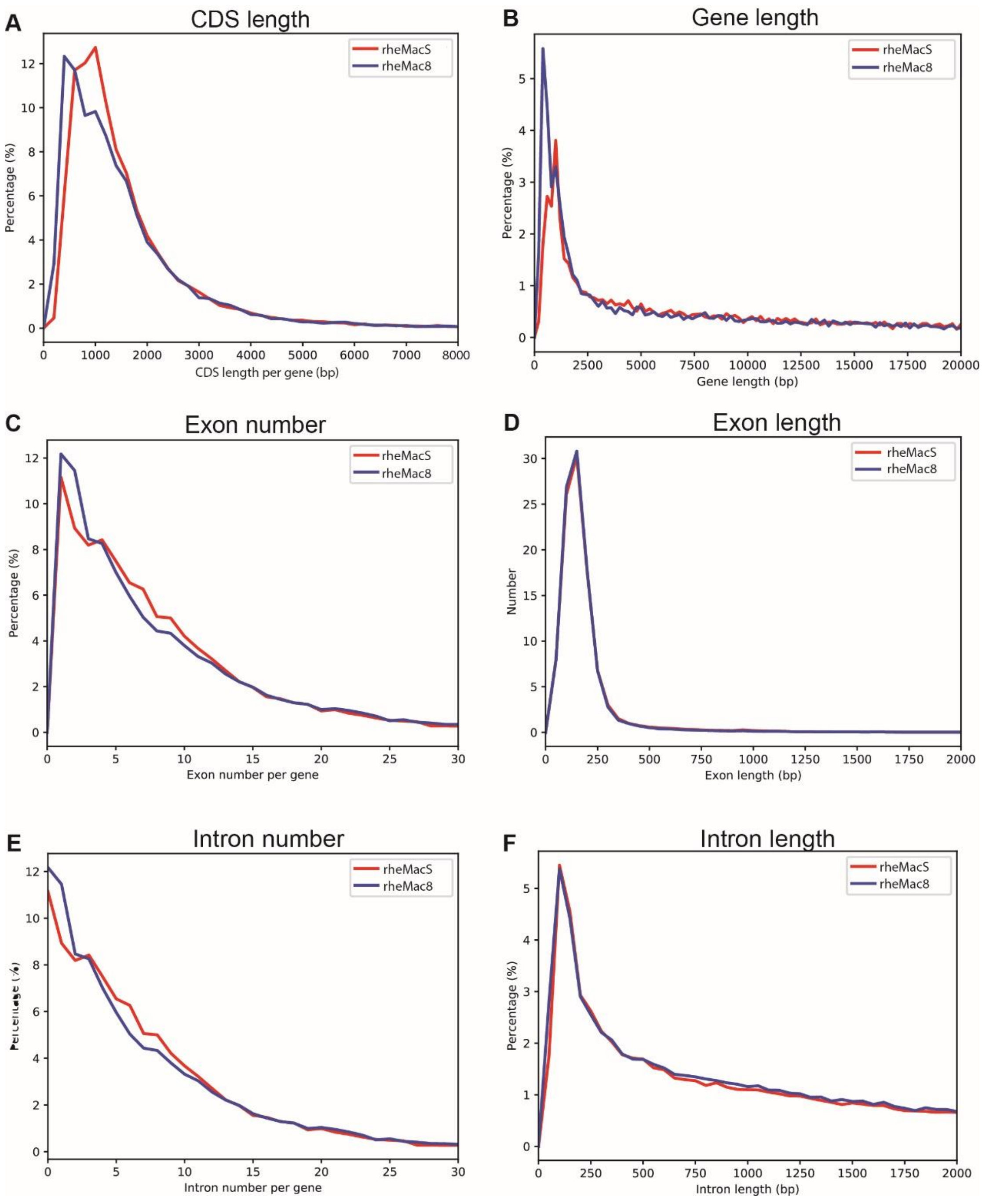

Supplementary Figure 8. Comparison of gene structures between rheMacS and rheMac8.

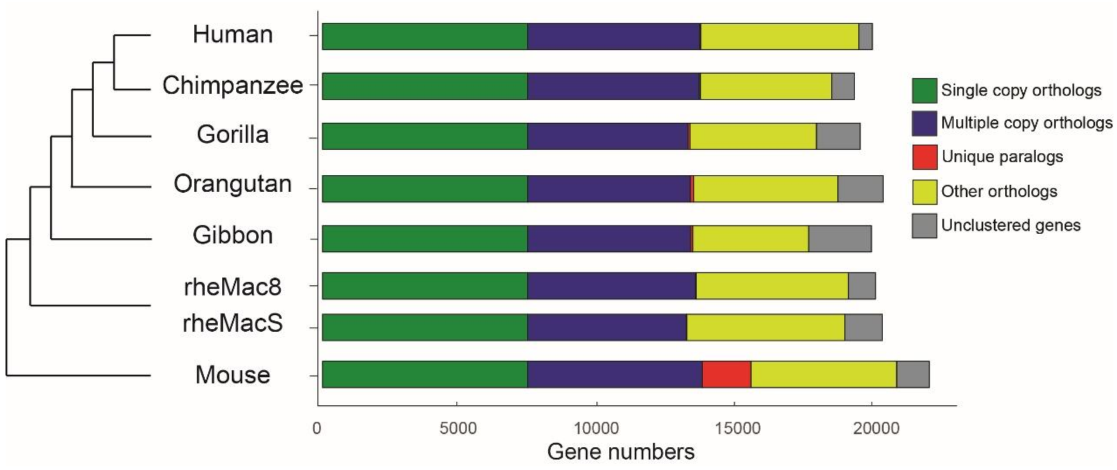

Supplementary Figure 9. Comparison of orthologous gene families among rheMacS, rheMac8 and genome assemblies of other species.

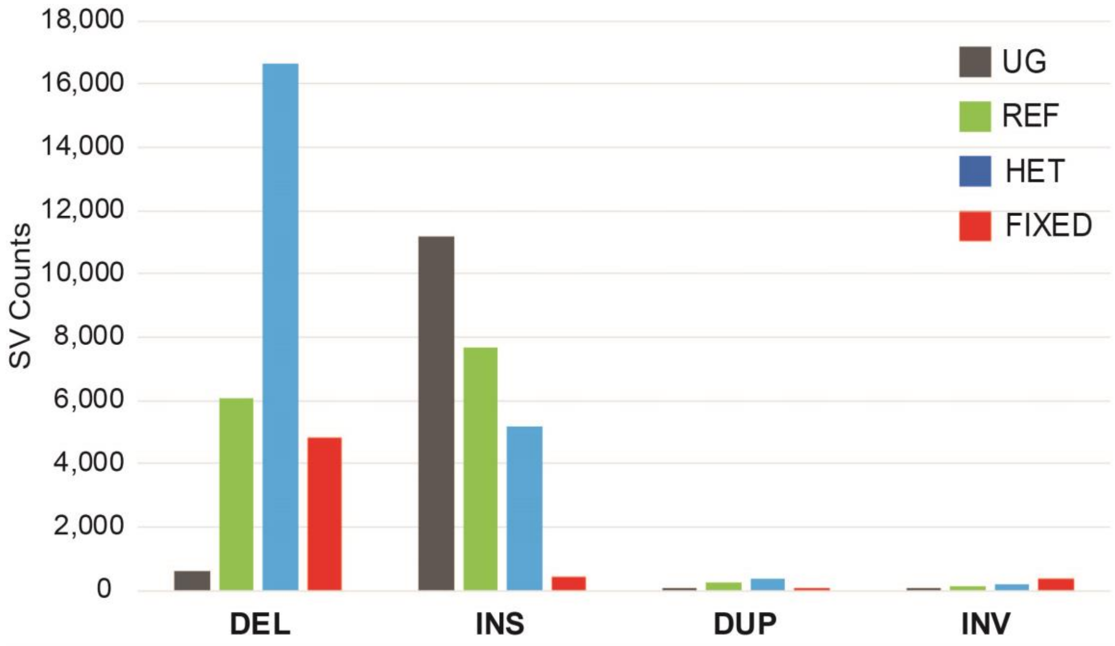

Supplementary Figure 10. Genotyping results of 53,916 SVs of rheMacS in five unrelated Chinese rhesus monkeys. *UG*: un-genotyped SVs; *REF*: reference SVs; *HET*: heterozygous SVs; *FIXED*: fixed SVs.

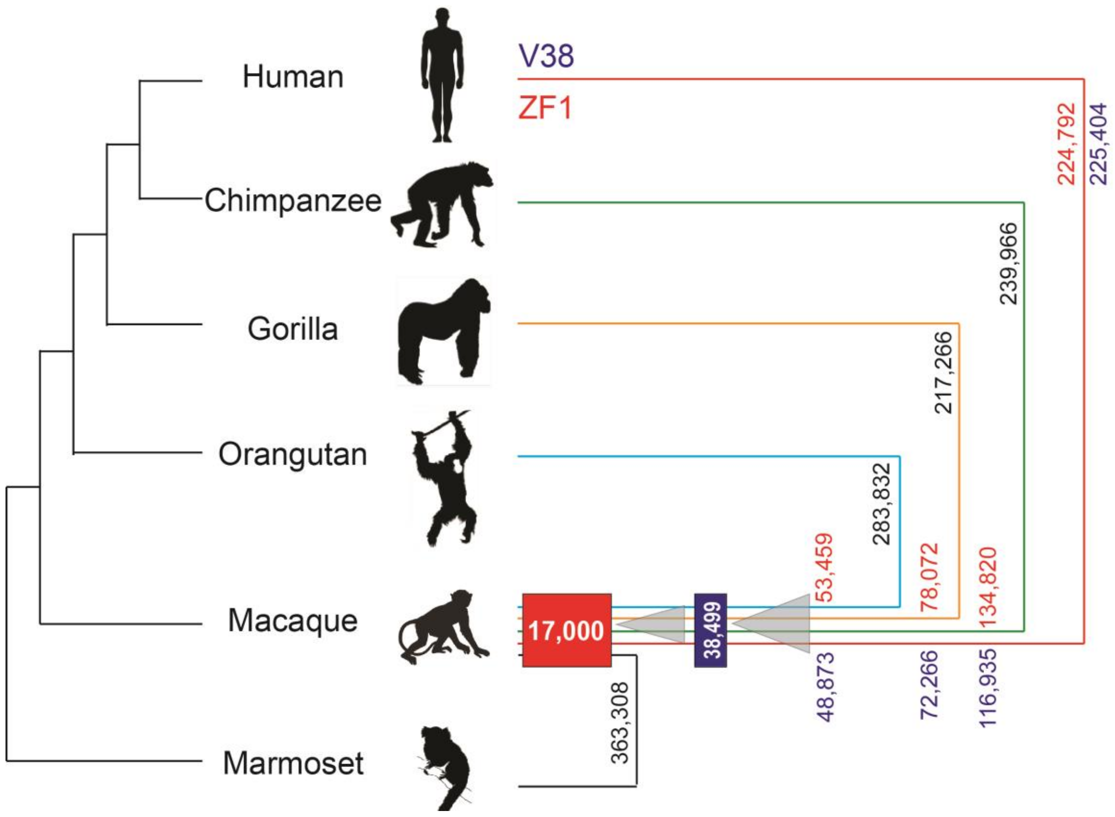

Supplementary Figure 11. Schematic diagram of ASSV calling. The ape and monkey cladogram show SVs assigned to lineages according to the assembly comparison. Gibbon is excluded due to its poor assembly quality. The SV count is shown on each branch. For human genomes, a long-read assembled genome ZF1 and the reference genome GRCh38 are used for SV calling, and the SVs number are marked in red (ZF1) and blue (V38). The common marmoset genome assembled by short-read sequencing is used to exclude the SVs that occurred in the monkey lineage. After obtaining the 38,499 SVs between macaque and apes, we then characterized SVs between macaque and marmoset and obtained 363,308 SVs. An SV is defined as an ape-specific structural variant or ASSV if it is included in the 38,499 set, but not in the 363,308 set. We obtained 17,000 ASSVs in total. The final set of ASSVs are highlighted in red box.

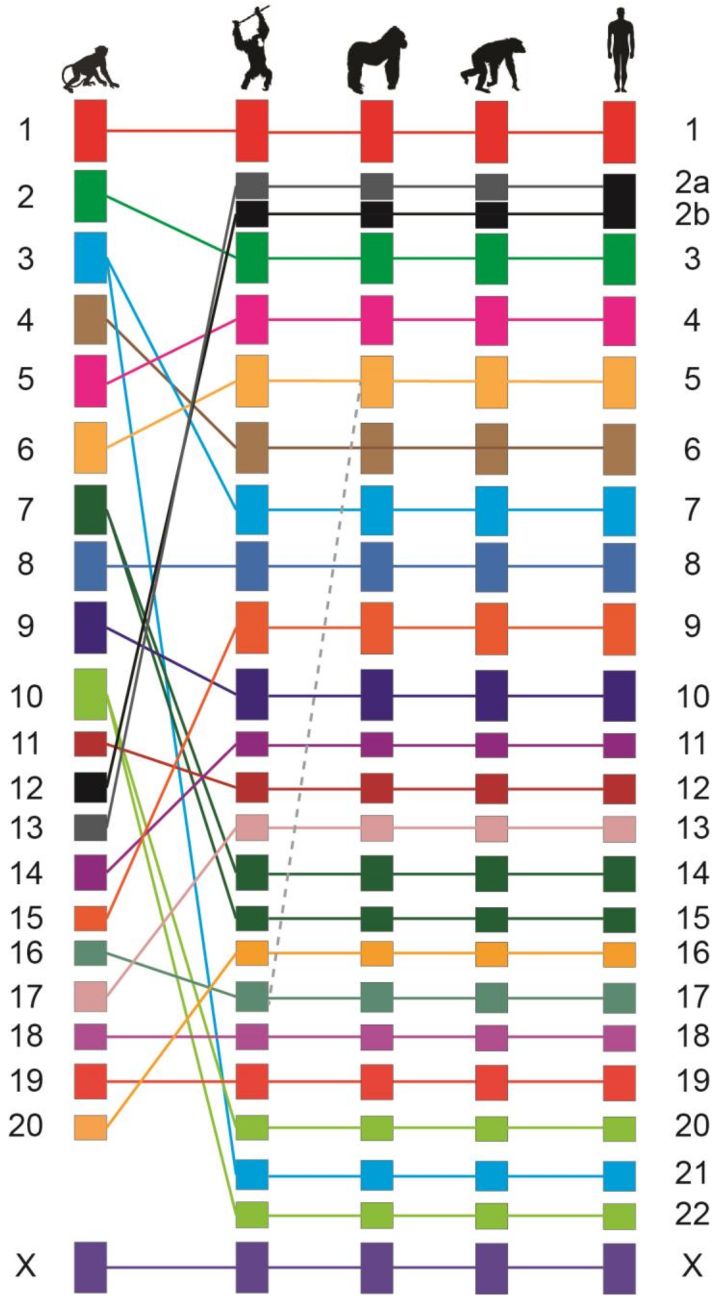

Supplementary Figure 12. The orthologous locations of ASSVs on each chromosome between rhesus macaque and apes are consistent with the known chromosomal synteny. The dashed line refers to the known translocation event between chromosome 17 and chromosome 5 in gorilla.

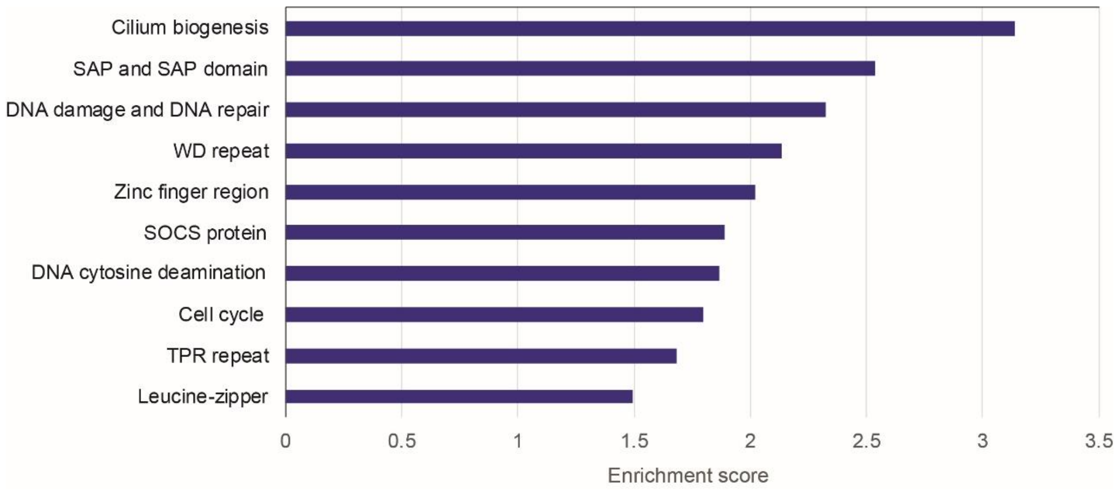

Supplementary Figure 13. Functional enrichment of the 3,412 ASSV-related genes. The top 5% (10/208) categories are shown.

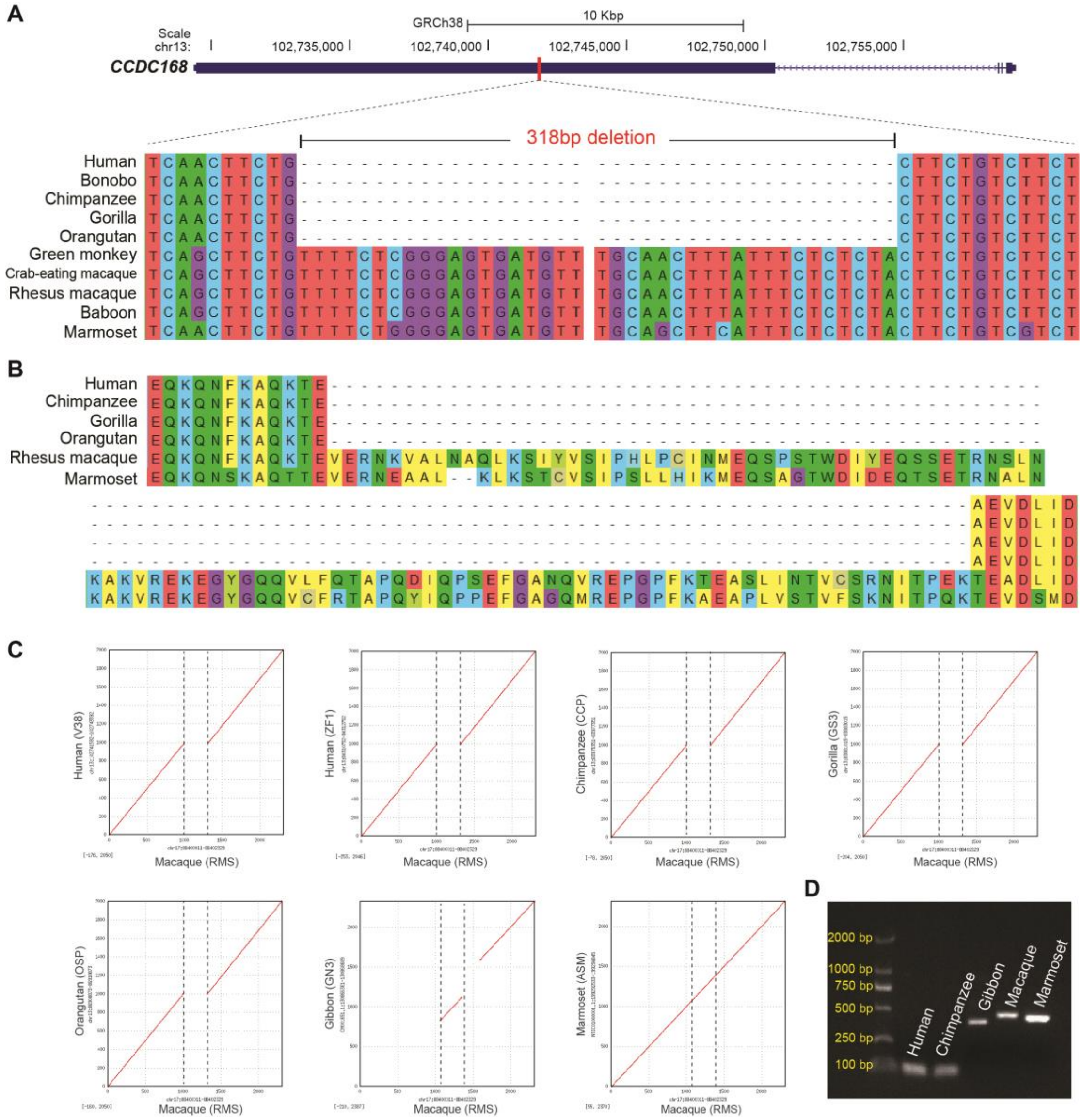

Supplementary Figure 14. An ASSV located in gene-coding regions. (A) A 318 bp deletion located in *CCDC168* in the great ape lineage. The graph shows multiple comparative alignments of the 318 bp deletion region. **(B)** Amino-acid alignments. The 318 bp coding-region deletion leads to a 106-amino acid deletion in the ape lineage. **(C)** Dot plots for pairwise comparison of the 318 bp deletion region (1 Kbp downstream and upstream flanking sequences) between macaque and apes. **(D)** PCR validation for 318 bp deletion in *CCDC168*.

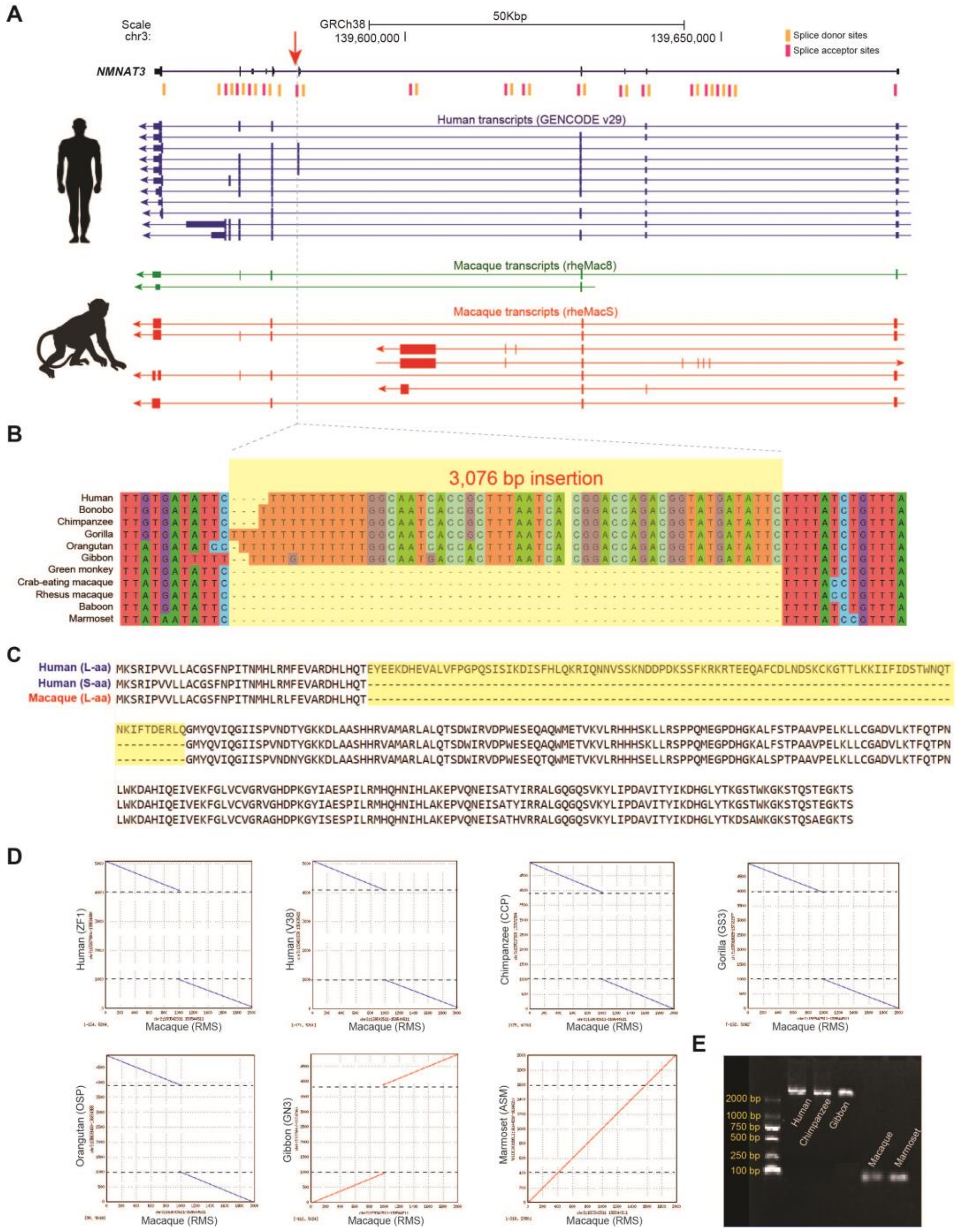

Supplementary Figure 15. A 3,076 bp insertion located in the splice acceptor of *NMNAT3* in the ape lineage. (A) The genomic location and transcript isoform comparison of *NMNAT3* between human and macaque. **(B)** Multiple comparative alignments of the 3,076 bp insertion region among primates. **(C)** Amino-acid alignment of *NMNAT3* between human and macaque. A human-specific protein isoform (highlighted) is identified, which is caused by the 3,076 bp insertion. **(D)** Dot plot for pairwise comparison of the insertion region (1 Kbp downstream and upstream flanking sequences) between macaque and apes. **(E)** PCR validation for 3,076 bp insertion in *NMNAT3*.

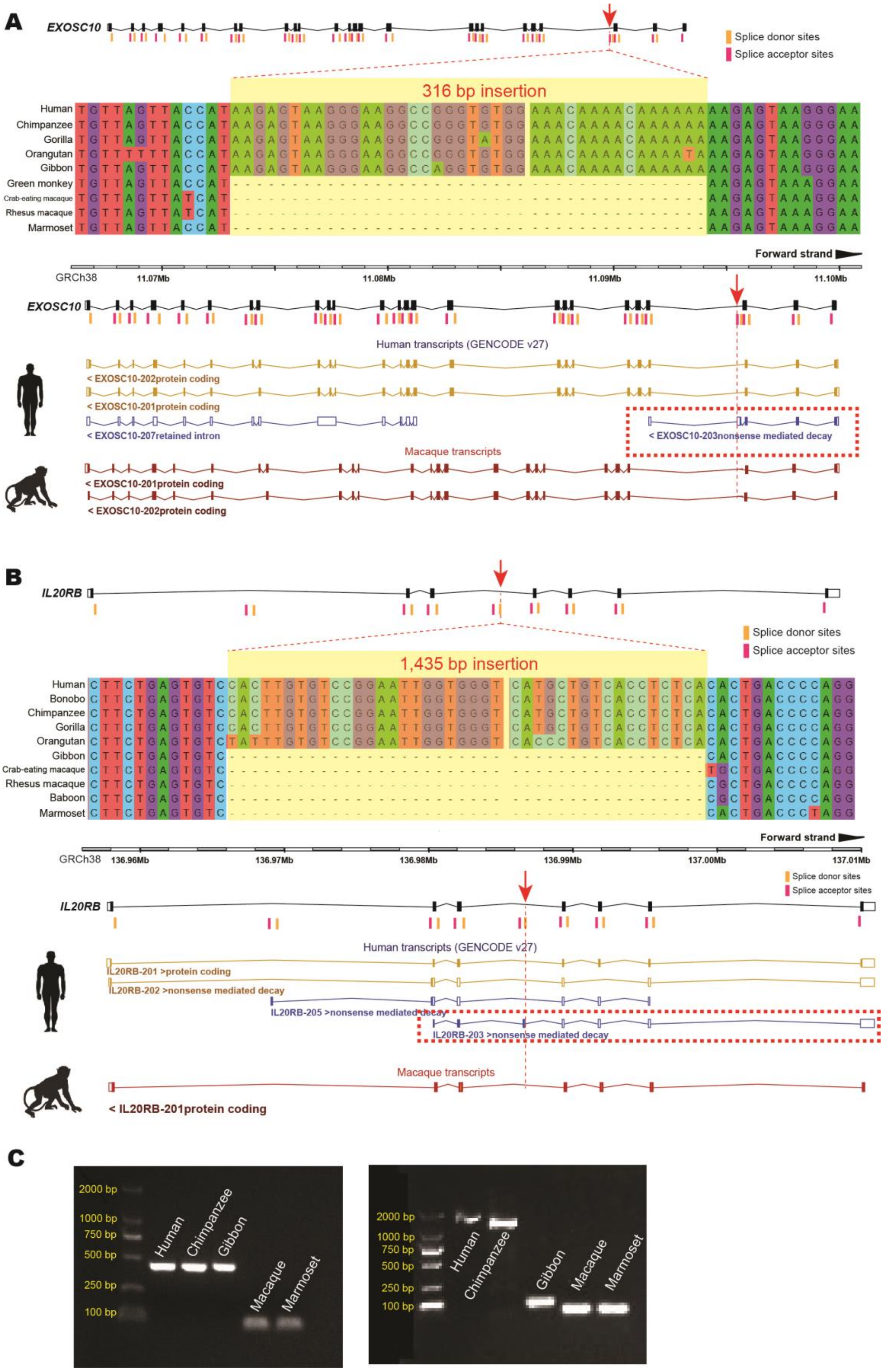

Supplementary Figure 16. Two ASSVs located at the splice sites of *EXOSC10* and

IL20RB. **(A)** A 316 bp insertion located in the splice acceptor of *EXOSC10*, resulting in a nonsense mediated decay (NMD) transcript (dashed frame) in the human lineage. The genomic location and multiple comparative alignments of the 316 bp insertion region (up-panel); transcript comparison of *EXOSC10* between human and macaque. **(B)** A 1,435 bp insertion located in the splice donor of *IL20RB*, resulting in an NMD transcript (dashed frame) in the human lineage. The genomic location and multiple comparative alignments of the 1,435 bp insertion region (up-panel); transcripts comparison of *IL20RB* between human and macaque. **(C)** PCR validations of 316 bp insertion in *EXOSC10* (left) and 1,435 bp insertion in *IL20RB* (right).

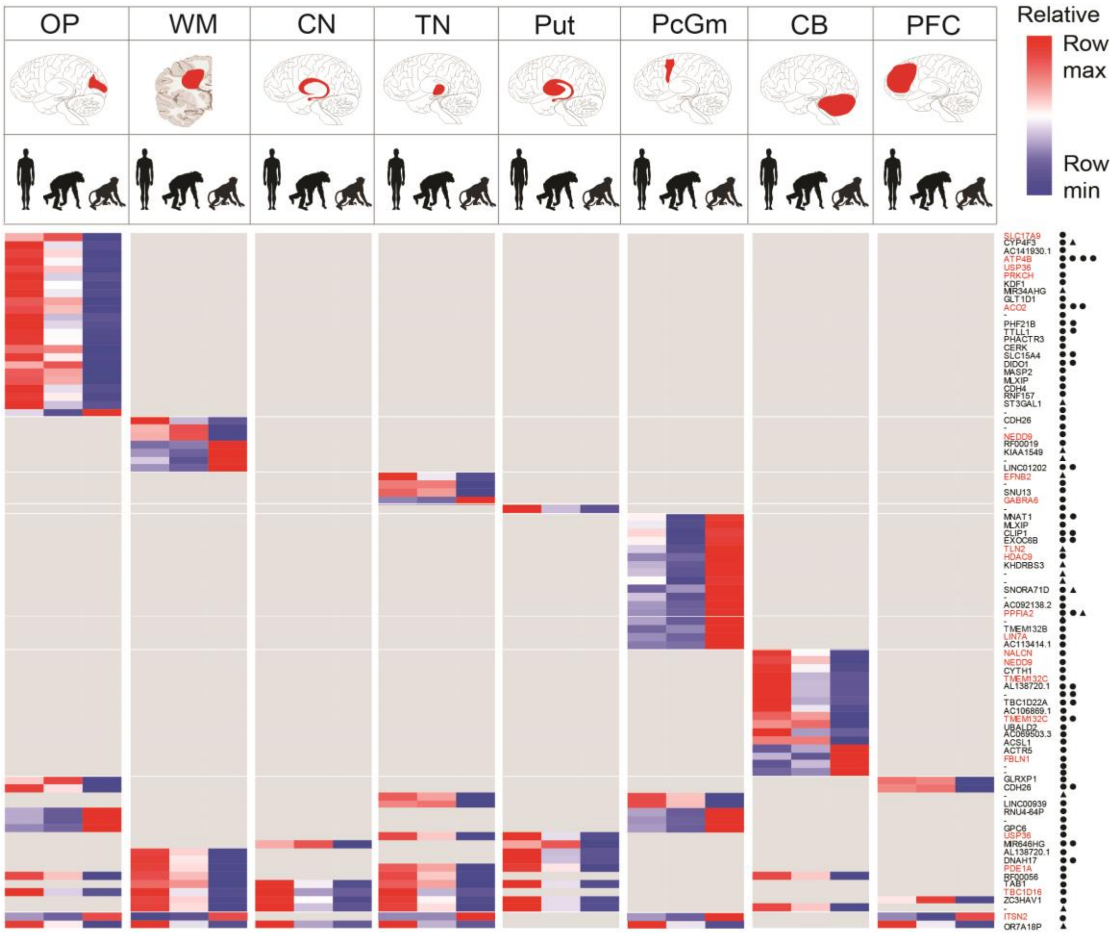

Supplementary Figure 17. Heatmap for ADEs with candidate ASSVs in eight brain regions. There are 87 ADEs (111 candidate ASSVs). The nearest genes are indicated and the corresponding brain regions are shown in red. The neuro-function-related genes are highlighted (red). ASSV deletions (circles) and insertions (triangles) are denoted.

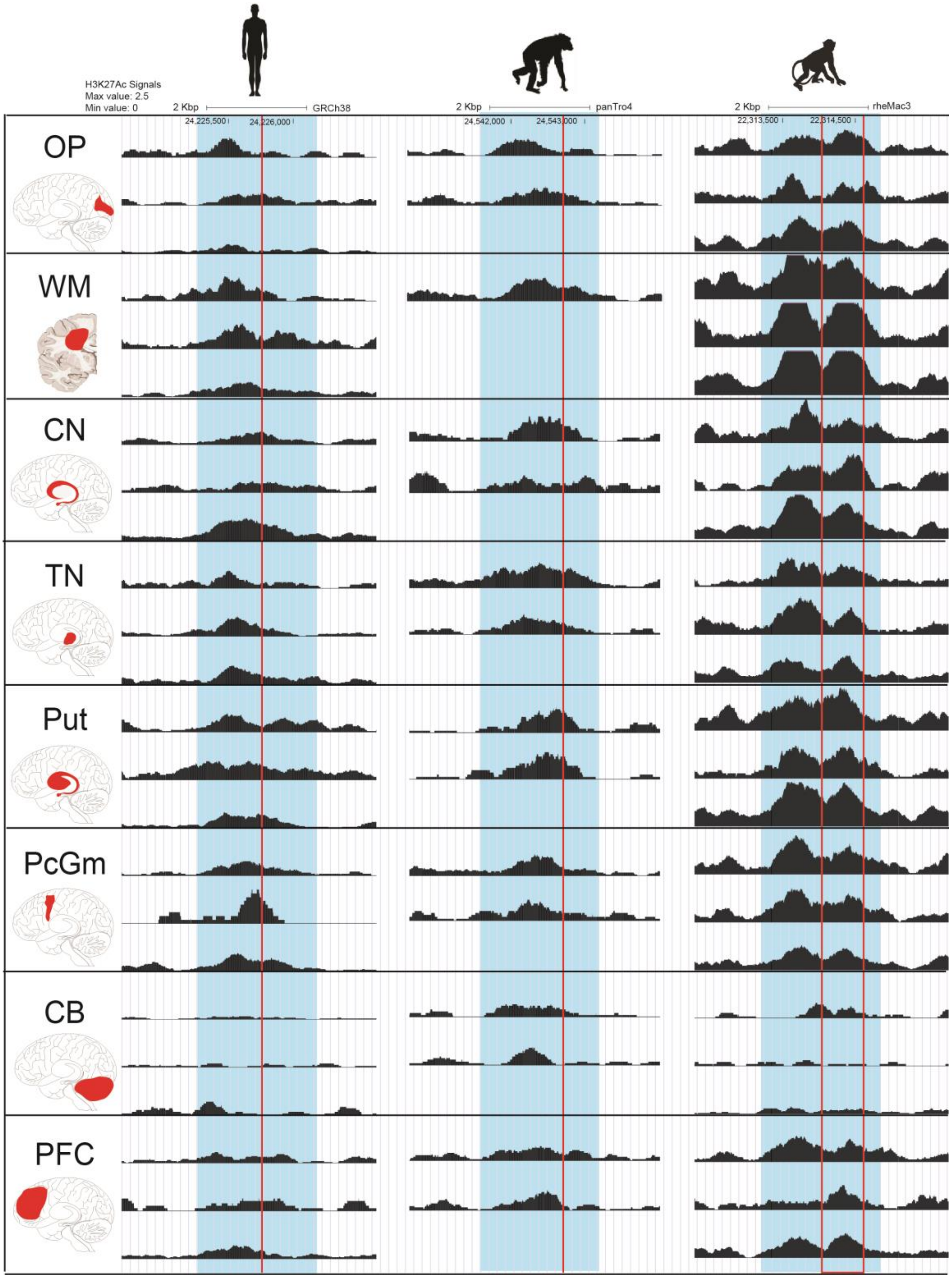

Supplementary Figure 18. Comparison of H3K27Ac signals of the ADE between apes and macaques, which possess an ASSV (587 bp deletion) in *ITSN2*. It shows that the ADE exhibits significant difference between apes (human: n=3 and chimpanzee: n=2) and macaque (n=3) in five brain regions. Shadow in light blue refers to ADE region, vertical lines and box in red refer to the ASSV region.

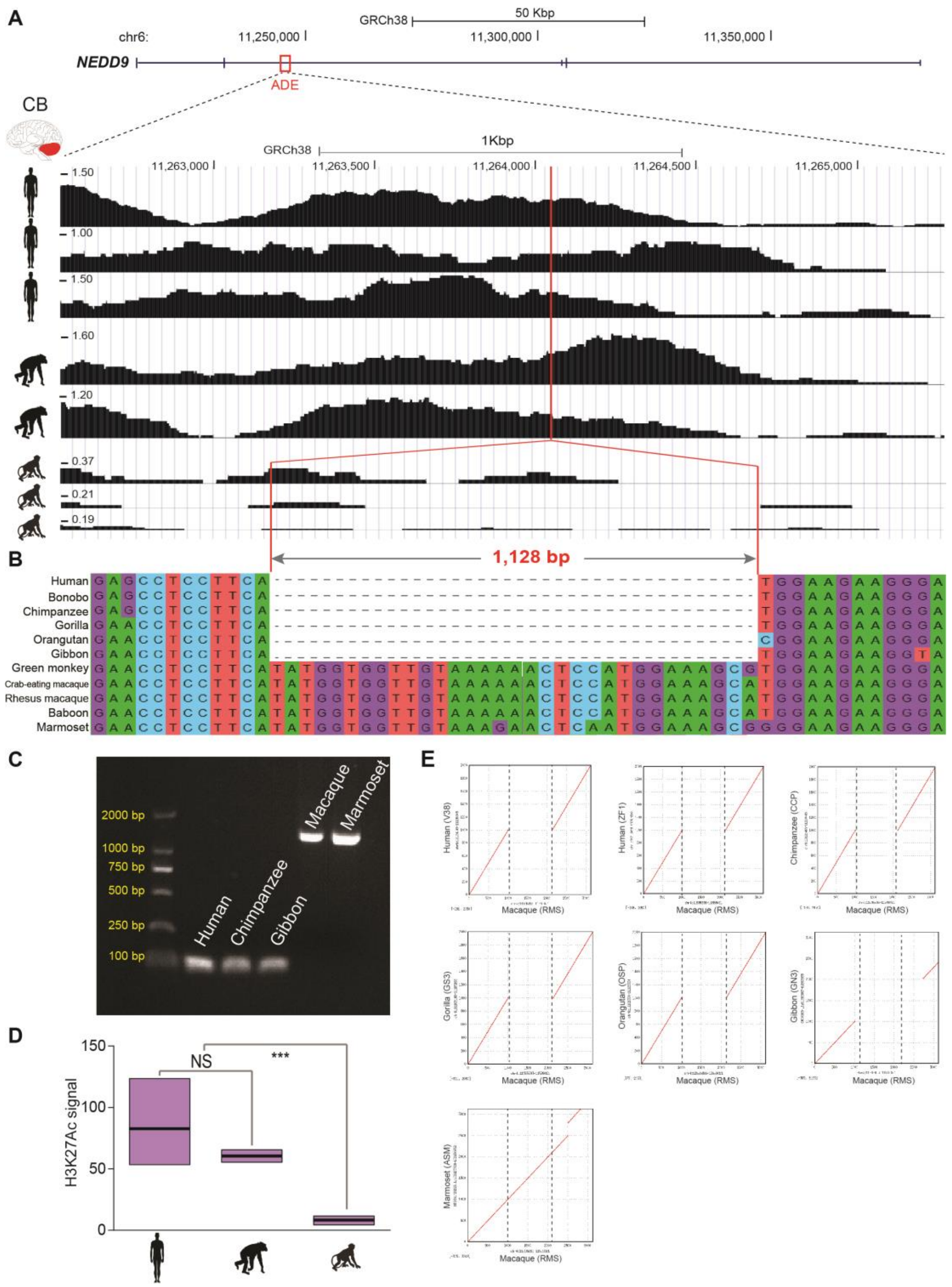

Supplementary Figure 19. A 1,128 bp deletion located at an ADE of *NEDD9* in the ape lineage. **(A)** Location of the 1,128 bp deletion in *NEDD9* and the H3K27Ac signals among human, chimpanzee and macaque. **(B)** Sequence alignment of the deletion region among apes and macaque. **(C)** PCR validation. **(D)** Comparison of the H3K27Ac signals among human, chimpanzee and macaque. (***-P<0.001; NS-not significant, P>0.05) **(E)** Dot plots for pairwise comparison of the 1,128 bp deletion region (1 Kbp downstream and upstream flanking sequences) between macaque and apes.

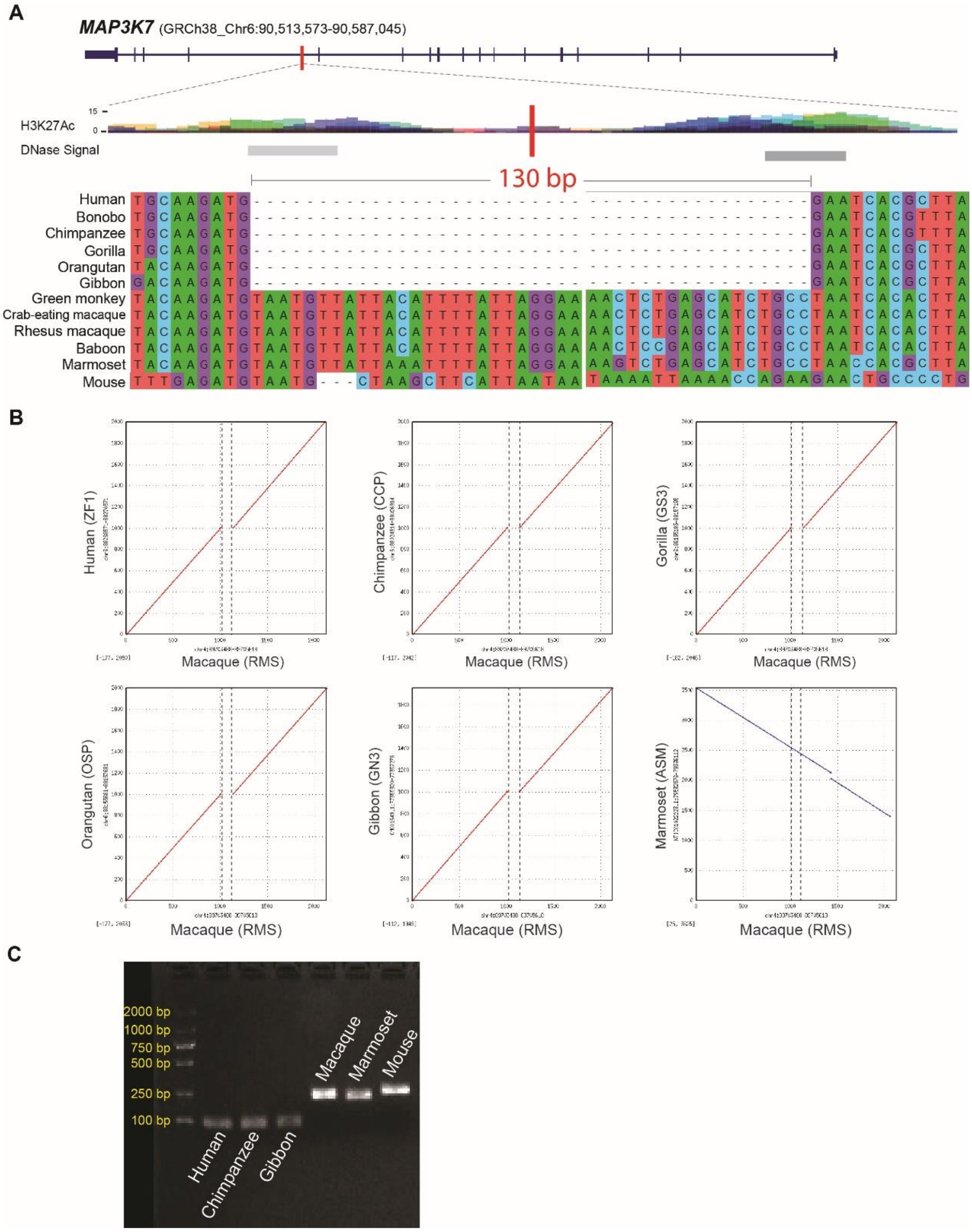

Supplementary Figure 20. An ASSV (130 bp deletion) located in a tail development related gene *MAP3K7*. (A) Location of the 130 bp deletion of *MAP3K7* in apes and sequence alignments. **(B)** Dot plot for the pairwise comparison of the 130 bp deletion region (1 Kbp downstream and upstream flanking sequences) between macaque and apes. **(C)** PCR validation of the 130 bp deletion.

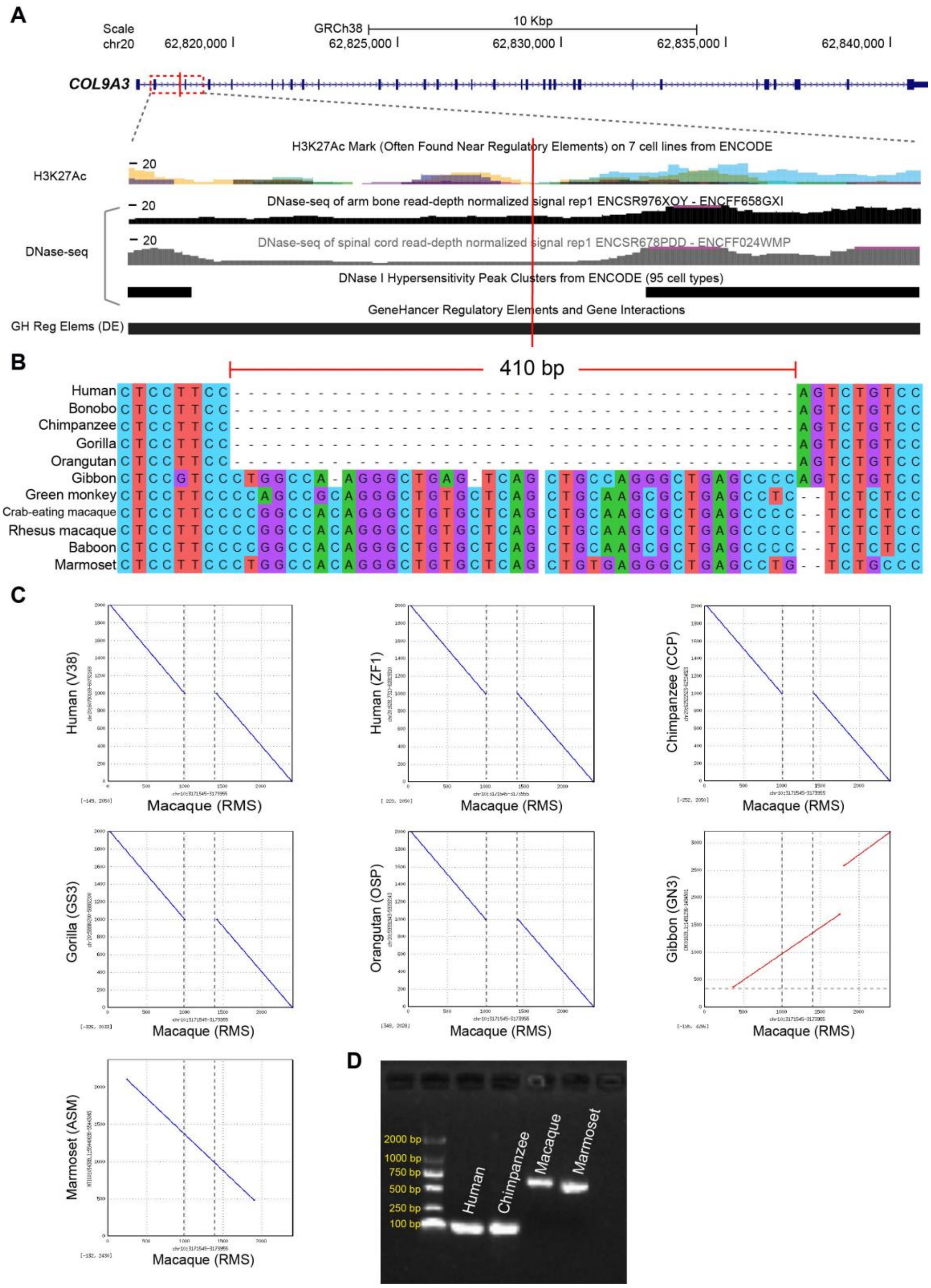

Supplementary Figure 21. A GASSV (410 bp deletion) located in the intron region of *COL9A3*, a gene related to body size. **(A)** Location of the 410 bp deletion in great apes and the gene regulatory annotations (from ENCODE). **(B)** Sequence alignments among primates. **(C)** Dot plot for the pairwise comparison of the deletion region (1 Kbp downstream and upstream flanking sequences) between macaque and apes. **(D)** PCR validation of the 410 bp deletion in *COL9A3*.

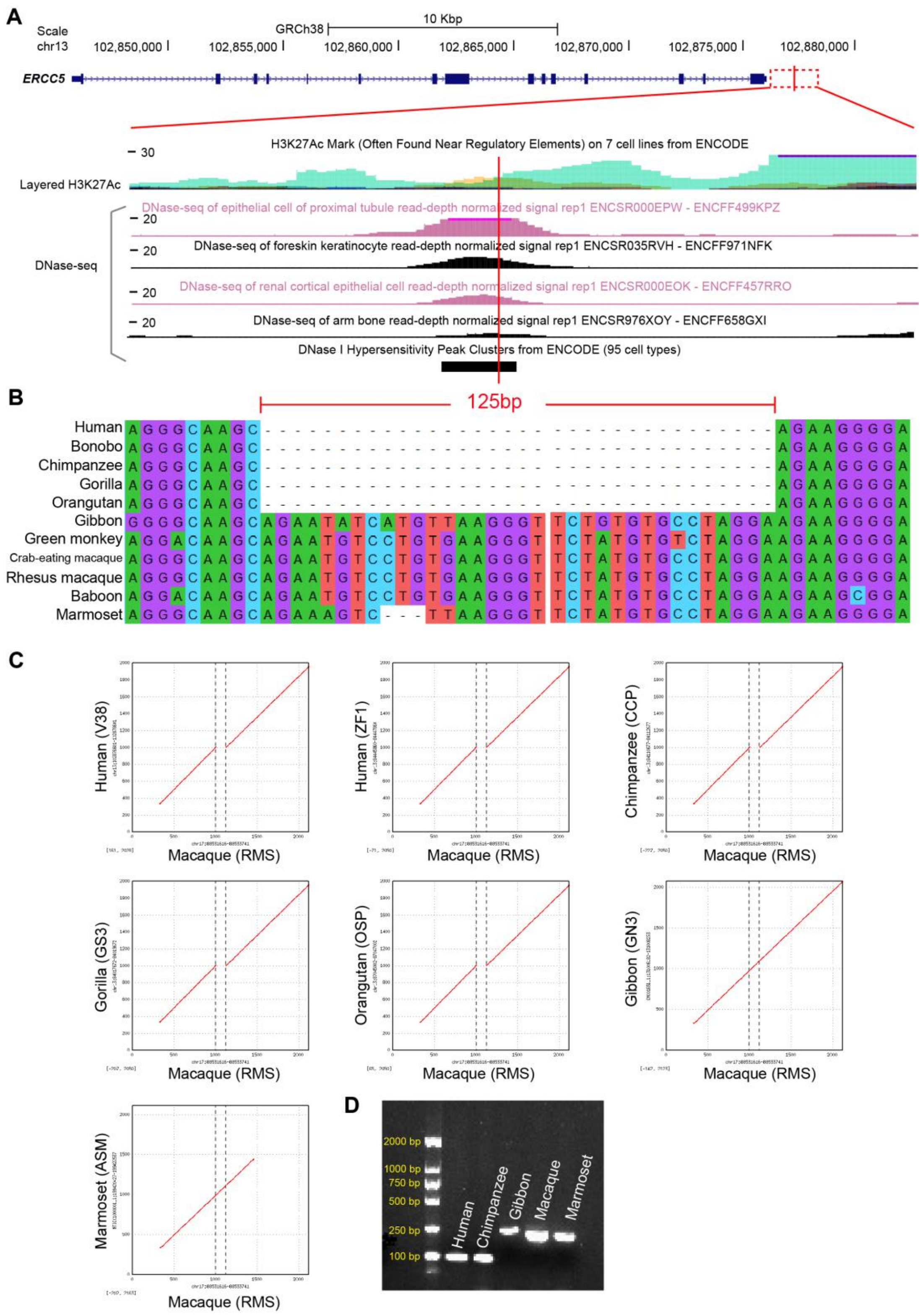

Supplementary Figure 22. A GASSV (125 bp deletion) located in the regulatory region of *ERCC5*, a gene related to body size. (A) Location of the 125 bp deletion in great apes and regulatory annotations (from ENCODE). **(B)** Sequence alignments among primates. **(C)** Dot plot for the pairwise comparison of the deletion region (1 Kbp downstream and upstream flanking sequences) between macaque and apes. **(D)** PCR validation of the 125 bp deletion in *ERCC5*.

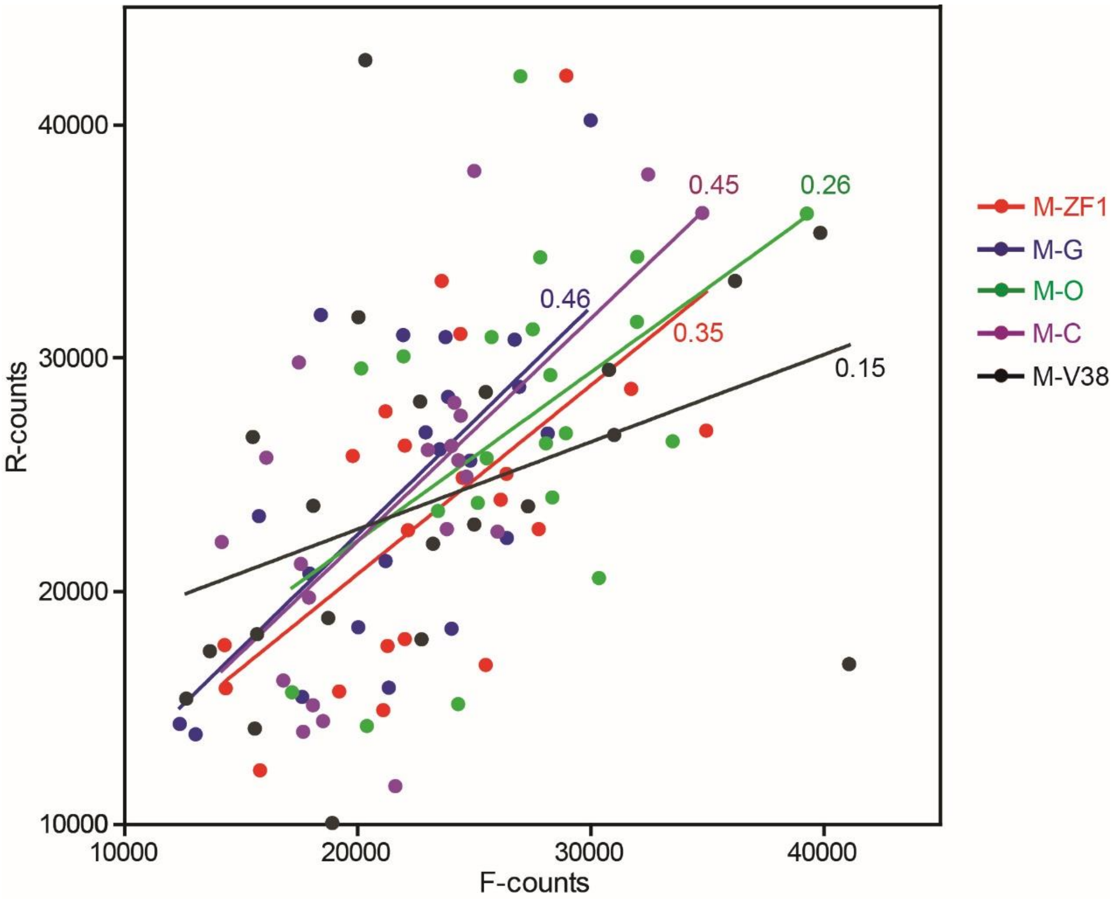

Supplementary Figure 23. Correlation of the SV numbers between forward-calling and reverse-calling. Genome-comparison-based SV callings are performed (Methods) between macaque (M) and apes, including ZF1-human (a long-read assembly of human), G-gorilla (GS3), O-Orangutan (OSP), C-chimpanzee (CCP) and V38-human (the human reference genome GRCh38). The SV counts are marked in different colors. Each dot refers to the different chromosome. The regression coefficients (R^2) are indicated.

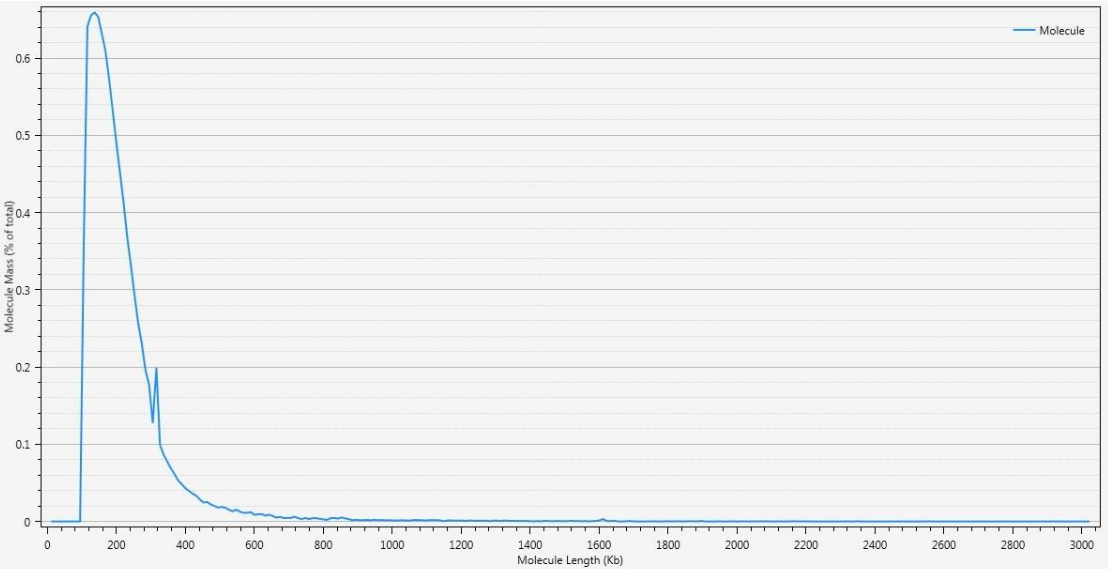

Supplementary Figure 24. Distribution of molecular length for the Bionano clean data.

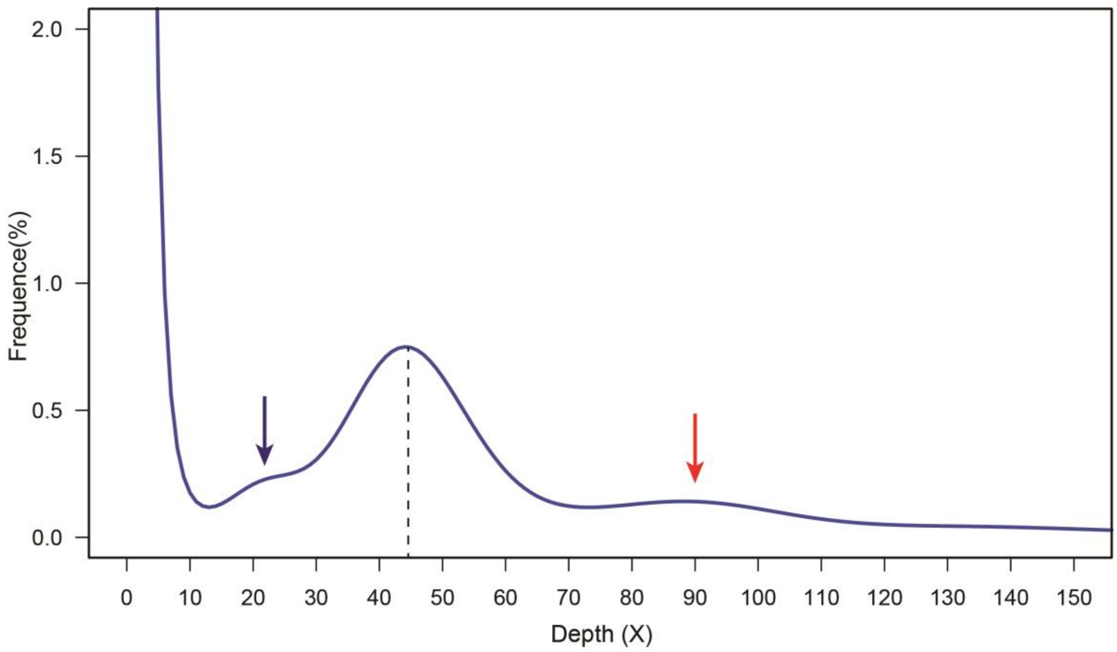

Supplementary Figure 25. The 17-mer depth distribution curve. K-mer depth is 45× (main peak: MP, vertical dashed line). Two secondary peaks (arrows) at X=1/2*MP and 2*MP coordinates, respectively, suggesting that rheMacS possess a high heterozygosity (arrow in blue) and high repeats (arrow in red).

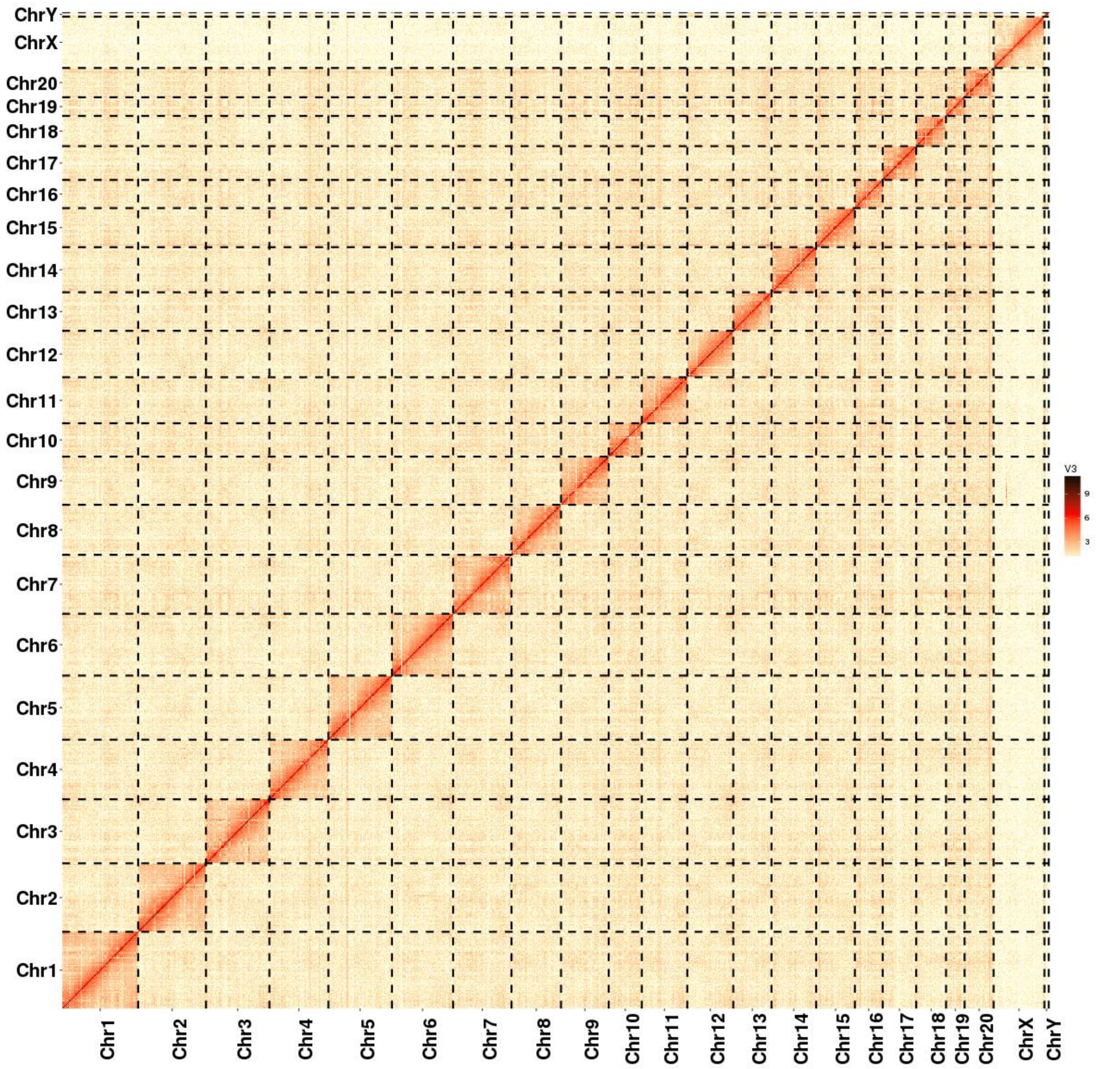

Supplementary Figure 26. Genome-wide all-by-all chromosome heatmap of the Hi-C data aligned to the rheMacS chromosomes.

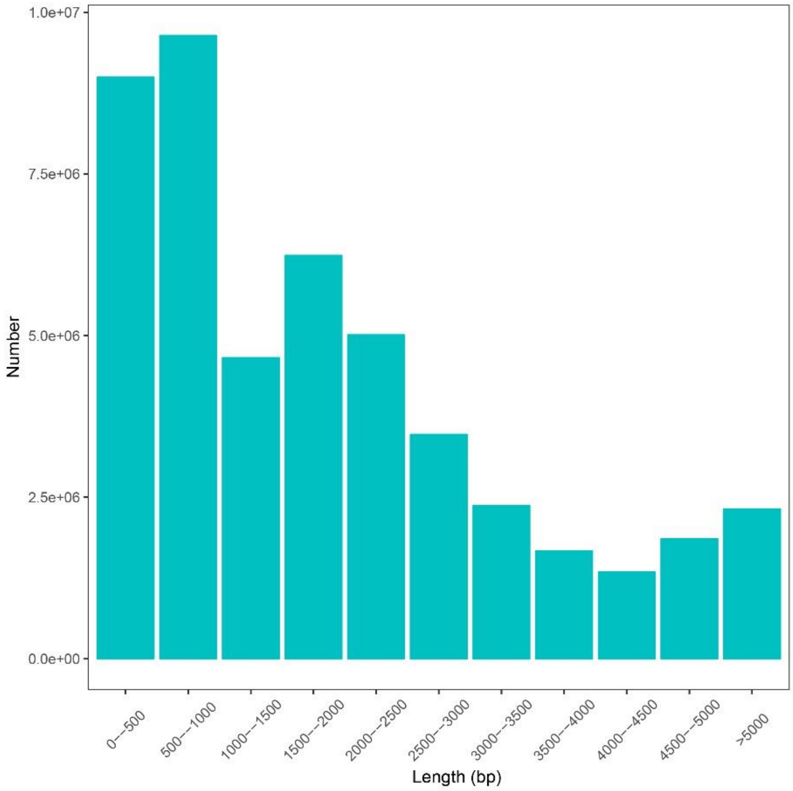

Supplementary Figure 27. Distribution of the subread lengths of the rheMacS Iso-Seq data.

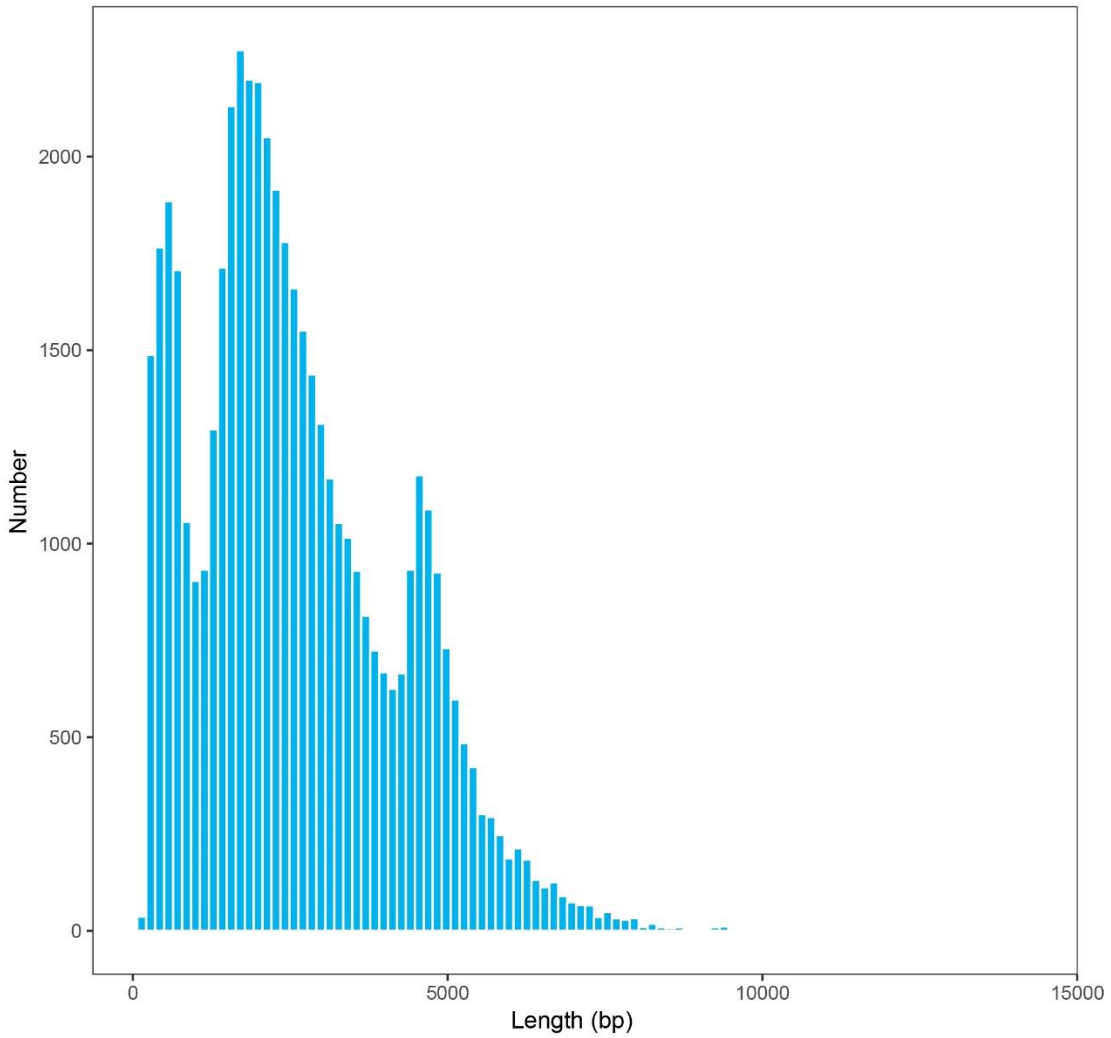

Supplementary Figure 28. Distribution of the lncRNA length of rheMacS.

Supplementary Tables

Supplementary Table 1. Data summary for this study.

Data	Sequencer	DNA resource	Depth	Read length	Total data (G)
PacBio	Sequal	Blood	100×	~9.7 Kbp	299.6
Illumina	X10	Blood	50×	150 bp	162
Bionano	Saphyr	Blood	101×	~8.6※	304
Hi-C	X10	Fibroblast	103×	150 bp	308
Iso-Seq	Sequal	multi-tissues*	-	2.8 Kbp	100
RNA-seq	X10	multi-tissues§	-	150 bp	185

*: 10 tissues: Heart, liver, spleen, lung, kidney, stomach, muscle, brain (PFA), cerebellum, testis
§: 16 tissues: large intestine, lung, epididymis, liver, testis, muscle, bladder, PFC, skin, spleen, kidney, stomach, small intestine, cerebellum, heart, pancreas.
※: label per 100Kbp.

Supplementary Table 2. Statistics of multiple assembled data.

	Stat Type	Contig Length （bp）	Contig Number	Scaffold Length （bp）	Scaffold Number
**FALCON**	N50	4,753,079	158	-	-
	N90	771,613	669	-	-
	Longest	33,382,844	1	-	-
	Length>=5kb	3,002,382,905	4,917	-	-
	Total	3,003,076,638	5,134	-	-
**Bionano**	N50	4,691,831	161	9,744,742	87
	N90	759,524	677	1,265,461	378
	Longest	33,382,844	1	49,445,782	1
	Length>=5kb	3,002,320,278	4,943	3,008,819,668	4,560
	Total	3,003,014,011	5,160	3,009,513,401	4,777
**PBJelly gap filling**	N50	8,096,761	100	13,609,070	61
	N90	1,100,637	444	1,634,467	282
	Longest	45,328,419	1	64,745,855	1
	Length>=5kb	3,023,027,334	4,544	3,025,829,092	4,336
	Total	3,023,694,962	4,751	3,026,496,720	4,543
**Arrow adjusted**	N50	8,187,147	99	13,638,801	61
	N90	1,106,151	439	1,637,744	283
	Longest	45,426,273	1	64,880,689	1
	Length>=5kb	3,030,843,238	4,536	3,033,643,665	4,338
	Total	3,031,501,491	4,741	3,034,301,918	4,543
**NGS data polished**	N50	8,187,147	99	13,638,801	61
	N90	1,106,151	439	1,637,744	283
	Longest	45,426,273	1	64,880,689	1
	Length>=5kb	3,030,843,238	4,536	3,033,643,665	4,338
	Total	3,031,501,491	4,741	3,034,301,918	4,543

Supplementary Table 3. Statistics of chromosome-level assembled using Hi-C data.

**Draft**	Total scaffolds	4,543
	Total scaffolds length (bp)	3,034,495,472
**Clustering**	Total scaffolds	2,684
	Proportion in total scaffolds (%)	59.08
	Total scaffold length of scaffolds (bp)	2,952,828,605
	Proportion of scaffold length in total length (%)	97.31
**Ordering and orienting**	Total scaffolds	2,684
	Proportion in total scaffolds (%)	100
	Total scaffold length of scaffolds (bp)	2,952,828,605
	Proportion of scaffold length in total length (%)	100

Supplementary Table 4. Summary of gaps and filled gaps in rheMac8 by rheMacS.

Chromosome	rheMacS		rheMac8		Filled gaps in rheMac8	Total length	Annotation			
							Repeat	Function		
	Gap number	Gap length (bp)	Gap number	Gap length (bp)				Exon	Intron	Intergenic
Chr1	119	332097	3949	5462089	1867	1541510	1531	0	662	1298
Chr2	126	150886	2908	5229210	780	4095409	611	2	467	585
Chr3	126	437641	3207	4755371	928	11231745	776	4	467	667
Chr4	186	436804	2623	4914955	914	2097959	734	3	333	620
Chr5	87	249253	2586	5681984	906	1946801	756	1	278	584
Chr6	108	176006	2536	4675540	603	944459	469	3	330	468
Chr7	145	201289	2964	3958247	453	4968412	332	3	539	332
Chr8	176	193645	2066	3782360	877	1225108	776	2	239	624
Chr9	219	381455	2004	3057750	740	1489258	622	1	322	514
Chr10	78	260712	2038	1705315	1350	1931718	984	1	363	912
Chr11	136	216499	2486	3937035	1509	1670980	1043	3	427	1071
Chr12	196	301052	1721	3731779	1174	586020	904	1	240	855
Chr13	154	244648	1771	2354883	1189	853721	878	2	230	939
Chr14	90	221197	1979	3120660	1180	946690	984	2	300	853
Chr15	124	227786	1949	2420094	1431	994657	1013	1	328	951
Chr16	106	154902	2100	2037118	943	4815881	743	2	371	722
Chr17	131	246193	1415	2670670	971	4399014	720	0	141	680
Chr18	163	166407	929	1769440	939	2240027	793	2	117	643
Chr19	71	365595	2502	1467830	1161	962532	997	6	368	772
Chr20	122	156949	1632	1748847	809	1130777	662	1	279	618
ChrX	135	283211	2515	3468578	1214	10659414	1042	1	302	908
ChrY	60	60060	2	100002	2	75769	1	0	2	1
**Total**	2858	5464287	47882	72049757	21940	60807861	17371	41	7105	14794
								21940		

Supplementary Table 5. 41 filled gaps interacted with exons in rheMac8 by rheMacS.

rheMac8 information			rheMacS information					
**Chrom**	**Start**	**End**	**Chrom**	**Start**	**End**	**Length (bp)**	**Gene ID**	**Gene Symbol**
chr2	50874271	50874297	chr2	49677422	49678035	613	Macaca.07932-RA	*PLXND1*
chr2	89978933	89979388	chr2	88469107	88469448	341	Macaca.19169-RA	*PPM1M*
chr3	36379599	36379620	chr3	36310345	36310575	230	Macaca.10369-RA	*KIF19*
chr3	12681166	12681192	chr3	13083532	13084016	484	Macaca.06098-RA	*PPIA*
chr3	40197809	40197830	chr3	40436743	40436766	23	Macaca.10756-RA	*GRID2IP*
chr3	149866119	149866140	chr3	154328722	154328783	61	Macaca.16344-RA	*r3*
chr4	98119128	98119149	chr4	98892363	98892620	257	Macaca.14164-RA	*PRDM13*
chr4	32699085	32699111	chr4	34291706	34292059	353	Macaca.04827-RA	*HSPA1*
chr4	43097732	43097753	chr4	44904905	44904907	2	Macaca.10570-RA	*C6orf132*
chr5	77708777	77708798	chr5	78418499	78418523	24	Macaca.17721-RA	*REST*
chr6	98686575	98686596	chr6	99989780	99989830	50	Macaca.19403-RA	*CTAGE15*
chr6	170969385	170969406	chr6	173689402	173689519	117	Macaca.09283-RA	*NA*
chr6	630213	630234	chr6	933247	933409	162	Macaca.09100-RA	*NA*
chr7	95876589	95877154	chr7	97944361	97945076	715	Macaca.13115-RA	*HNRNPC*
chr7	157758871	157758892	chr7	144424251	144424399	148	Macaca.12846-RA	*SYNE3*
chr7	69077218	69078067	chr7	70979517	70979828	311	Macaca.03402-RA	*UNC45A*
chr8	16707186	16707207	chr8	17629235	17629304	69	Macaca.00703-RA	*MICU3*
chr8	116588881	116589318	chr8	119532937	119533272	335	Macaca.18717-RA	*FAM133B*
chr9	63736731	63737033	chr9	77117055	77117422	367	Macaca.05565-RA	*STOX1*
chr10	19268240	19268266	chr10	20785602	20786114	512	Macaca.10804-RA	*SEMG2*

chr11	7437943	7438747	chr11	7639036	7639915	879	Macaca.00328-RA	*C1S*
chr11	36744458	36744479	chr11	39466973	39466988	15	Macaca.05086-RA	*HERVK_113*
chr11	22605505	22605507	chr11	23575051	23575053	2	Macaca.05445-RA	*ABCC9*
chr12	63130587	63130608	chr12	70402074	70402427	353	Macaca.00050-RA	*HOXD11*
chr13	87180048	87180069	chr13	86458273	86458403	130	Macaca.01673-RA	*SH2D6*
chr13	45690208	45690229	chr13	45439090	45439384	294	Macaca.18887-RA	*SIX3*
chr14	16873643	16873664	chr14	17974350	17974367	17	Macaca.11450-RA	*Olfr1030*
chr14	32211864	32211885	chr14	33055812	33055893	81	Macaca.11578-RA	*C11orf91*
chr15	27060631	27060652	chr15	30097913	30098040	127	Macaca.02577-RA	*PTBP3*
chr16	57619278	57619299	chr16	61638705	61638847	142	Macaca.02266-RA	*PRR29*
chr16	4466922	4467121	chr16	4687007	4687968	961	Macaca.13950-RA	*GGT6*
chr18	46890126	46890147	chr18	56801620	56801631	11	Macaca.00850-RA	*ONECUT2*
chr18	11630061	11630510	chr18	15717937	15718683	746	Macaca.13543-RA	*CABLES1*
chr19	39880610	39880631	chr19	40869705	40869803	98	Macaca.04259-RA	*PVR*
chr19	39880610	39880631	chr19	40869705	40869803	98	Macaca.04258-RA	*PVR*
chr19	1577748	1578223	chr19	1660373	1660994	621	Macaca.11620-RA	*ONECUT3*
chr19	17482613	17483313	chr19	17654108	17654260	152	Macaca.14748-RA	*FAM129C*
chr19	31647995	31648017	chr19	32269422	32269424	2	Macaca.02796-RA	*KMT2B*
chr19	43865203	43865224	chr19	44875849	44875950	101	Macaca.19101-RA	*LMTK3*
chr20	34563526	34563547	chr20	37088966	37089002	36	Macaca.01875-RA	*NKD1*
chrX	2090280	2090301	chrX	3384624	3384754	130	Macaca.18258-RA	*ARSF*

Supplementary Table 6. Repeat analysis of filled gaps for rheMac8.

Repeat elements		Count	Average	Standard	Total	Percent
DNA		286	135.591	124.778	38,779	7.181
LINE	CR1	7	320.286	238.113	2,242	0.176
	I	3	45.000	21.517	135	0.075
	I-Jockey	2	34.500	16.263	69	0.050
	L1	969	1,492.235	1,890.571	1,445,976	24.32
	L1-Tx1	1	33.000	0.000	33	0.025
	L2	123	180.545	187.796	22,207	3.088
	Penelope	2	41.000	19.799	82	0.050
	R1	0	NA	NA	NA	NA
	R2	1	40.000	0	40	0.025
	Rex	1	83.000	0	83	0.025
	RTE	3	114.667	122.647	344	0.075
Low		14	119.214	24.370	1669	0.351
LTR	Copia	1	37.000	0	37	0.025
	DIRS	0	NA	NA	NA	NA
	ERV1	71	730.704	1,011.284	51,880	1.783
	ERVK	12	185.500	257.986	2,226	0.301
	ERVL	201	243.090	260.651	48,861	5.046
	ERVL-MaLR	85	220.412	126.773	18,735	2.134
	Gypsy	20	117.050	75.577	2,341	0.502
	non	134	2,908.246		389,705	3.364
RC	Helitron	6	78.500	27.399	471	0.151
Retroposo	SVA	67	99.149	95.874	6,643	1.682
Satellite	acro	3	142.667	79.387	428	0.075
	centr	2	203.500	3.536	407	0.050
	non	14	156.286	94.499	2,188	0.351
	telo	0	NA	NA	NA	NA
	Y-chromosome	0	NA	NA	NA	NA
Simple		883	792.270	10,759.26	699,574	22.16
SINE	Alu	898	244.157	117.122	219,253	22.54
	MIR	119	119.798	55.677	14,256	2.988
	tRNA-7SL	8	32.125	25.554	257	0.201
	tRNA-Deu	1	206.000	0.000	206	0.025
	7SL	16	38.938	20.879	623	0.402
Unknown		30	179.167	278.797	5,375	0.753

Supplementary Table 7. Statistics of assembled chromosomes

Chromosome	Length(bp)	Consistency with rheMac8 (%)
Chr1	227,617,462	98.70
Chr2	203,273,356	98.64
Chr3	189,974,636	98.72
Chr4	177,000,330	98.70
Chr5	190,508,718	98.64
Chr6	183,305,545	98.54
Chr7	175,094,158	98.66
Chr8	148,403,059	98.61
Chr9	142,822,578	98.77
Chr10	98,845,734	98.87
Chr11	136,270,821	98.71
Chr12	137,731,144	98.76
Chr13	114,312,528	98.90
Chr14	133,980,097	98.74
Chr15	116,006,700	98.70
Chr16	83,193,081	98.87
Chr17	100,348,525	98.75
Chr18	89,280,157	98.86
Chr19	55,557,796	98.59
Chr20	86,713,556	98.86
ChrX	152,195,021	98.28
ChrY	13,055,603	92.14
Total	2,955,490,605	98.41

Supplementary Table 8. Statistics of SNVs calling by NGS reads mapped to rheMacS assembly.

CHR	Homozygous*		Heterozygous		Base error rate	QV§
	SNPs	INDELs	SNPs	INDELs		
1	586	1,958	409,987	87,845	1.1177E-05	50
2	436	1,525	377,290	80,593	9.6471E-06	50
3	418	1,791	360,867	79,254	1.1628E-05	49
4	545	1,528	346,611	74,574	1.1712E-05	49
5	359	1,517	390,748	85,285	9.8473E-06	50
6	452	1,301	346,872	74,756	9.5633E-06	50
7	431	1,440	315,745	66,215	1.0686E-05	50
8	469	1,250	272,645	58,046	1.1583E-05	49
9	674	1,374	255,053	53,545	1.4339E-05	48
10	300	944	179,363	35,988	1.2585E-05	49
11	385	1,276	248,785	53,908	1.2189E-05	49
12	326	1,011	243,045	52,391	9.7073E-06	50
13	400	919	203,625	42,696	1.1539E-05	49
14	291	1,059	236,280	51,135	1.0076E-05	50
15	299	1,021	201,263	43,003	1.1379E-05	49
16	294	906	140,540	30,393	1.4424E-05	48
17	320	821	208,902	45,775	1.1370E-05	49
18	302	694	156,999	34,263	1.1156E-05	50
19	279	1,089	94,092	20,434	2.4623E-05	46
20	339	817	154,434	30,863	1.3331E-05	49
X	140	581	6,639	1,425	4.7373E-06	53
Y	71	148	2,725	535	1.6774E-05	48
Total	8,116	24,970	5,152,510	1,102,922	1.1195E-05	50

*Homozygous SNVs represent the site with both of the alleles that are different from the nucleotide on the rheMacS assembly.

§QV (quality value) represents a per-base. Estimate of accuracy and is calculated as QV = -10log10(P) where P is the probability of error.

Supplementary Table 9. Summary of five macaques Illumina WGS reads mapped to rheMac8 and rheMacS.

	Sample_ID	ind01	ind02	ind03	ind04	ind05	Average
	Reads(R1)	466857306	470771424	470068763	470697093	437754192	46322
	Reads(R2)	466857306	470771424	470068763	470697093	437754192	46322
	Q30(R1)	6609135886	6681546654	6649554276	6665289515	6001294348	6521364136
	Q30percent(R1)	94.3776724	94.6184115	94.3061214	94.4030972	91.3951932	93.8200992
	Q30(R2)	6247352153	6312226386	6103149202	6283342157	5315916498	6052397279
	Q30percent(R2)	89.2114438	89.3884103	86.5568286	88.9934277	80.9574048	87.0215030
	meanQ30percent	91.7945581	92.0034109	90.4314750	91.6982624	86.1762990	90.4208011
rheMac8	rawDepth	49.33	49.79	48.96	49.71	45.81	48.72
	eDPmean*	43.88	44.72	42.88	43.54	40.1	43.024
	PercentDup(%)	11.21	10.36	12.69	12.67	12.82	11.95
	Unmapped	7287928	7256120	7439753	6833332	7300353	7223497.2
	mapped	935067171	942772195	941199697	943290162	876917653	92784
	Mapped rate(%)	0.992	0.992	0.992	0.993	0.992	0.992
	ProperlyPaired(	96.29	96.34	96.14	96.21	96.15	96.226
	ISmedian§	414	417	400	422	400	410.6
	ISmean	421.942201	424.746374	406.829866	429.041836	403.416798	417.195415
	ISsd	103.434228	104.774544	101.548837	107.607837	96.347229	102.742535
rheMacS	rawDepth	46.63	47.07	46.3	46.98	43.391858	46.0743716
	eDPmean	43.27	44.19	43.24	43.89	41.644	43.2468
	PercentDup(%)	0.072005	0.061006	0.066035	0.065704	0.06645925	0.06624185
	Unmapped	3936674	3830300	3910183	3577036	387 0,633.8	382
	mapped	935541266	943259959	942025822	943845904	87568	92807
	Mapped rate(%)	0.99580972	0.99595571	0.99586633	0.99622445	0.99560587	0.99589242
	ProperlyPaired(	97.6	97.64	97.33	97.34	97.2755	97.4371
	ISmedian	415	418	401	423	401	411.6
	ISmean	423.504241	426.444567	408.631068	431.100473	405.066882	418.949446
	ISsd	103.102195	104.395091	101.313405	107.697082	95.6837696	102.438308

*: eDP-effective depth; §: IS-insert size.

Supplementary Table 10. Summary of detected variants by rhesus Illumina WGS data.

Variant type		Tools	Reference based on	
			**rheMac8**	**rheMacS**
SNVs	SNPs	GATK	19,474,353	19,172,495
		Samtools	18,661,340	19,124,913
	INDELs	GATK	4,523,318	3,957,728
		Samtools	4,823,333	5,615,821
SVs	Deletions	Delly	147,931	135,218
	Insertions	Delly	35,067	33,823
	Duplications	Delly	42,512	44,246
	Inversions	Delly	46,629	33,691
	Total		24,269,810	23,377,201

Supplementary Table 11. Iso-seq subreads mappablity for rheMacS and rheMac8.

Items	rheMac8	rheMacS
Total number	1,267,315	1,267,862
Total mapped number	1,255,469	1,257,845
Total mapped rate	0.9907	0.9921
Number of identity filtered	133,686	125,534
Number of coverage filtered	434,978	406,455
Total filter rate	0.4581	0.4275
Mean consensus number per isoform	2.48	2.51
Collapse isoforms	276,600	288,773

Total number: the total Iso-seq transcripts number filtered fusion genes

Total mapped number: the total number of mapped Iso-seq transcripts

Total mapped rate: the proportion of mappped Iso-seq transcripts for total number

Number of identity_filtered: the number of Iso-seq transcripts passed identity filtering

Number of coverage_filtered: the number of Iso-seq transcripts passed coverage filtering

Total filter rate: the proportion of filtered Iso-seq transcripts for total number

Mean consensus number per isoform: the average number of consensus sequence in an isoform

Number of collapse isoforms: the isoform number after pruning redundance

Supplementary Table 12. Comparison of gene statistics of rheMacS and other species.

Specie	Version	Genes	Gene length	CDS length	Exons	Exon length	Intron length
Human	GRCh38.p12	20,022	50,541.15	1,722.17	9.88	174.27	5,496.22
Chimp	Clint_PTRv2	18,269	48,278.78	1,619.01	9.83	164.77	5,286.83
Gorilla	gorGor4	20,920	40,770.71	1,513.78	9.27	163.38	4,749.58
Orangutan	P_pygmaeus_2.0.2	20,424	45,357.04	1,490.71	9.56	155.91	5,123.82
Gibbon	Nleu_3.0	19,978	47,585.41	1,531.21	9.39	163.10	5,490.26
Macaque	rheMac8	20,605	45,285.37	1,489.06	8.89	167.48	5,550.02
Macaque	rheMacS	20,389	44,276.53	1,564.38	8.88	176.24	5,422.77
Mouse	GRCm38.p6	22,094	38,075.20	1,563.10	8.82	177.15	4,666.83

Genes: total number of gene

Gene length: average gene length (bp)

CDS length: Average CDS length (bp)

Exon: Average exons number per gene

Exon length: Average exon length (bp)

Intron length: Average intron length (bp)

Supplementary Table 13. Summary of repeat content of rheMacS.

Terms	Macaque (RM8)	Macaque (RMS)	Orangutan (OSP)	Gorilla (GS3)	Chimpanzee (CCP)	Human (ZF1)
Total (%)	52.24	54.04	53.19	52.8	52.7	54.07
srpRNA (%)	0.01	0.01	0.01	0.01	0.01	0.01
LTR (%)	10.21	9.94	10.39	10.41	10.42	10.43
SSR (%)	1.16	1.18	1.02	1.05	1.01	1.22
SINE (%)	13.75	13.29	12.8	13.02	13.04	13.01
DNA (%)	4.05	3.89	4.11	4.13	4.13	4.1
Simple repeat (%)	1.2	1.22	1.21	1.23	1.19	1.96
Unknown (%)	0.05	0.05	0.05	0.05	0.05	0.05
scRNA (%)	0.01	0.01	0.01	0.01	0.01	0.01
snRNA (%)	0.01	0.01	0.01	0.01	0.01	0.01
rRNA (%)	0	0.01	0	0	0.01	0.01
tRNA (%)	0	0	0	0	0	0
RC (%)	0.02	0.02	0.02	0.02	0.02	0.02
LINE (%)	22.49	21.94	23.97	23.13	23.09	23.05
RNA (%)	0.01	0.01	0.01	0.01	0.01	0.01
Retroposon (%)	0	0	0.1	0.14	0.13	0.12
Low complexity (%)	0.21	0.22	0.21	0.20	0.20	0.21

Supplementary Table 14. Summary of BUSCO prediction.

Type	rheMacS		rheMac8	
	Number	Percent (%)	Number	Percent (%)
Complete BUSCOs	3,836	93.5	3,867	94.2
Complete and single-copy BUSCOs	3,745	91.3	3,842	93.2
Complete and duplicated BUSCOs	91	2.2	43	1
Fragmented BUSCOs	191	4.7	112	2.7
Missing BUSCOs	77	1.8	125	3.1
Total BUSCO groups searched	4,104	-	4,104	-

Supplementary Table 15. Statistics of ncRNA annotation of rheMacS.

Type		Counts	Average Length (bp)	Total Length (bp)	Percentage of genome (%)
rRNA	rRNA	544	168.54	91,685	0.001828
	18S	1	1,868.00	1,868	0.000037
	28S	4	7,414.75	29,659	0.000591
	5.8S	4	151.5	606	0.000012
	5S	535	111.31	59,552	0.001188
snRNA	snRNA	2,373	112.68	267,378	0.005332
	CD-box	496	96.41	47,820	0.000954
	HACA-box	379	131.67	49,903	0.000995
	splicing	1,498	113.25	169,655	0.003383
miRNA		11,035	247.37	2,729,689	0.054434
tRNA		718	73.67	52,894	0.001055
lncRNA		49,698	2,582.25	128,332,745	4.3421807

Supplementary Table 16. Information of assemblies used in this study.

Assembly name	Species	Common name	Database	Accession	Alias in this study	PacBio based
rheMacS	*Macaca mulatta*	Rhesus monkey	NCBI	PRJNA514196	RMS	TURE
rheMac8 (Mmul_8.0.1)	*Macaca mulatta*	Rhesus monkey	NCBI	PRJNA214746	RM8	FALSE
ZF1	*Homo sapiens*	Human	GSA	PRJCA000936	ZF1	TURE
GRCh38.p12	*Homo sapiens*	Human	NCBI	PRJNA31257	V38	FALSE
panpan1.1	*Pan paniscus*	Bonobo	NCBI	PRJNA49285	-	FALSE
Clint_PTRv2	*Pan troglodytes*	Chimpanzee	NCBI	PRJNA369439	CCP	TURE
Pan_tro 3.0	*Pan troglodytes*	Chimpanzee	NCBI	PRJNA13184	-	FALSE
Susie3	*Gorilla gorilla gorilla*	Western lowland gorilla	NCBI	PRJEB10880	GS3	TURE
gorGor4	*Gorilla gorilla gorilla*	Western lowland gorilla	NCBI	PRJEA31265	-	FALSE
Susie_PABv2	*Pongo abelii*	Sumatran orangutan	NCBI	PRJNA369439	OSP	TURE
P_pygmaeus_2.0.2	*Pongo abelii*	Sumatran orangutan	NCBI	PRJNA20869	-	FALSE
Nleu_3.0	*Nomascus leucogenys*	Northern white-cheeked gibbon	NCBI	PRJNA13975	GN3	FALSE
Chlorocebus_sabeus 1.1	*Chlorocebus sabaeus*	Green monkey	NCBI	PRJNA168621	-	FALSE
Macaca_fascicularis_5.0	*Macaca fascicularis*	Crab-eating macaque	NCBI	PRJNA20409	-	FALSE
Panu_3.0	*Papio anubis*	Baboon	NCBI	PRJNA54005	-	FALSE
Callithrix jacchus-3.2	*Callithrix jacchus*	Common marmoset	NCBI	PRJNA20401	-	FALSE
ASM83236v1	*Callithrix jacchus*	Common marmoset	NCBI	PRJNA246742	ASM	FALSE
GRCm38 (p6)	*Mus musculus*	House mouse	NCBI	PRJNA20689	-	FALSE

Supplementary Table 17. Repeat annotation of ASSVs.

		Insertions			Deletions		
ASSV		3,544			13,456		
Total base		2,139,296			5,830,871		
Masked		1,614,579			4,700,799		
Percentage		75.47			80.62		
		number	length	percentage	number	length	percentage
SINEs:		2,126	470,389	21.99	10,074	2,366,221	40.58
	ALUs	1,966	450,009	21.04	9,813	2,332,824	40.01
	MIRs	160	20,380	0.95	260	33,312	0.57
LINEs:		1,080	677,585	31.67	2,017	1,534,500	26.32
	LINE1	933	645,236	30.16	1,800	1,493,075	25.61
	LINE2	136	30,030	1.4	200	39,329	0.67
	L3/CR1	9	1,493	0.07	12	1,651	0.03
LTR		578	386,355	18.06	1,056	577,699	9.91
	ERVL	73	25,585	1.2	137	49,418	0.85
	ERVL	195	63,345	2.96	301	76,188	1.31
	ERV	245	238,640	11.16	425	290,148	4.98
	ERV	57	57,095	2.67	180	159,179	2.73
DNA		219	42,877	2	311	55,328	0.95
	hAT	105	18,104	0.85	155	21,965	0.38
	TcMar	54	13,260	0.62	91	20,843	0.36
Unclassified		9	3,064	0.14	1	467	0.01
Small RNA		19	1,483	0.07	32	3,504	0.06
Satellites		18	1,486	0.07	2	530	0.01
Simple		483	28,221	1.32	2,169	145,832	2.5
Low		45	3,119	0.15	172	16,771	0.29

Supplementary Table 18. The SVs sequence synteny of each chromosome between rhesus monkey and apes.

RMS	ZF1	GS3	OP3	CCP	V38
chr1	chr1	chr1	chr1	chr1	chr1
chr2	chr3	chr3	chr3	chr3	chr3
chr3	chr7/21	chr7/21	chr7/21	chr7/21	chr7/21
chr4	chr6	chr6	chr6	chr6	chr6
chr5	chr4	chr4	chr4	chr4	chr4
chr6	chr5	chr5	chr5	chr5	chr5
chr7	chr14/15	chr14/15	chr14/15	chr14/15	chr14/15
chr8	chr8	chr8	chr8	chr8	chr8
chr9	chr10	chr10	chr10	chr10	chr10
chr10	chr20/22	chr20/22	chr20/22	chr20/22	chr20/22
chr11	chr12	chr12	chr12	chr12	chr12
chr12	chr2	chr2b	chr2B	chr2B	chr2
chr13	chr2	chr2a	chr2A	chr2A	chr2
chr14	chr11	chr11	chr11	chr11	chr11
chr15	chr9	chr9	chr9	chr9	chr9
chr16	chr17	chr/5chr17	chr17	chr17	chr17
chr17	chr13	chr13	chr13	chr13	chr13
chr18	chr18	chr18	chr18	chr18	chr18
chr19	chr19	chr19	chr19	chr19	chr19
chr20	chr16	chr16	chr16	chr16	chr16
chrX	chrX	chrX	chrX	chrX	chrX

Supplementary Table 19. Gene list for related to ASPs and GASPs.

Functions	Gene list*
Tail development	*ACAN, ADM, AHR, ANKRD13A, APC,* ***AXIN1****, BMP11, BMP2, BMP4, BMP5, BMPER, BMPR1A, BW19, CAT,* ***CDH8****, CDX1, CDX2, CDX4, CENPJ, CER1, CHUK, COL11A2, CREB1, CTNNB1, CYP26A1, CYP51A1, DACT1, DKK1, DLL1, DLL3, DMPK, DUSP4, DVL2, DVL3, EPHA2, ETN2, EVX1, EVX2, FGF10, FGF17, FGF3, FGF4, FGF8, FGF9, FGFR1, FGFR3, FOXA1, FOXA2, FZD7, G3BP1, GDF11, GFAP, GUSB, HBEGF, HES7, HLXB9, HMGA2, HOXA11, HOXA5, HOXB1, HOXB13, HOXB8, HOXC13, HOXD11, IGF2R, IHH, KTN1, LEF1, LEPR, LFNG, LMX1A, LRP6,* ***MAP3K7****, MEA1, MEOX1, MEOX2, MESP2, MNX1, MSGN1, MTA1, MTHFR, MUT, MYSM1, NOG, NOTCH1, NOTCH2,* ***NOTO****, NPPC, NPR2, NPR3, NRARP, PARD3, PAX1, PCSK5, PHEX, PLXND1, POFUT1, POR, PORCN,* ***PPP5C****, PSEN1, PTCH1, PTEN, PTF1A, PTK7, RARG, RIPPLY2, ROR2, RPL24, RPL38, RPS7,* ***SFN****, SFXN1, SHH, SLX4, SOX5, SOX6, SOX9, SP8, SULF1, SULF2, TACC3, TACR3, TBX6, TCF1, TCF15, TRIP13, TWSG1, VANGL1, VANGL2, VCL, WNT3A, WNT5A, WNT5B, XRCC2, ZBTB16, ZIC3*
Primary microcephaly	*MCPH1,* ***WDR62****, CDK5RAP2, CASC5, ASPM, CENP7, CPAP, CEP135, CEP152, ZNF335, PHC1, CDK6, CENPE, SASS6, MFSD2A, ANKLE2, CTT, WDFY3, ALFY,* ***TRNP1***
Adducted thumbs	*TPM2, LIFR, COL6A1, COL6A2, COL6A3, MYH3, SEMA3D, SEMA3C, LIMK1,* ***ERCC1****, UBA1, COL12A1, FBN1, FBN2, GTF2I, CRLF1, TAPT1, NUP107,* ***NALCN****, CHST14, CLIP2,* ***NRTN****, KCNH1, L1CAM, TPRKB, BAZ1B, ALDH18A1, ISPD, LAGE3, WDR73, ALG3, ECEL1, RBM8A, ELN, RET, TP53RK, AP4M1, OSGEP, RFT1, RFC2, TNNI2, ECE1, GTF2IRD1, TNNT3, ANKLE2, NEK9, GDNF, SLC9A6, TBL2, PHGDH, DSE, EDN3, EDNRB, ADGRG6, MYBPC1, ALG13*
Body size	*ACAN, ACTB, ADAMTS10, ANKRD11, ANKRD26, ARG, ARHGAP18, ARID1B, ARSE, ASN540SER, B4GALT7, BDLN1, BDLN3, BDLN4, BDLN5, BDLN6, BDNF, BGN, BMP2, BRF1, BTF2, BZW2, C5, CANT1,* ***CBX7****, CCDC8, CDMP1, CEP19, CGH , CHK1, CNP, COA3, COL11A2, COL1A1, COL2A1, COL9A2,* ***COL9A3****, COMP,* ***CPE****, CPHD, CREB, CRIPT, CRTL1, CRTM, CSA, CSB, CSH1, CTLP, CUL7, CYP21A2, DACHS, DDR2, DENND1B, DENV, DMBX1, DMM, DNAJB3, DPH1, DUOX2, DUOXA2, DVL3, DXYS15, EED, EIF2AK3, EPHA5,* ***ERCC2****, ERCC3,* ***ERCC5****, ERCC6, ERCC8, ESR1, ESR2, EXOSC2, FAHD2A, FAM111A, FBN1, FGD1,* ***FGF21****, FGFR3, FGL1, FMR1, FOXO1, GCG, GDF5, GH, GHR , GHRD3,* ***GHRH****, GHSR, GIMAP8, GLIS3, GMNN, GON4L, GPC3, GPD1, GPS, HESX1, HMGA2, HOS, HOXA5, HRAS, HSPB1, HTR3B, IARS2, IGF1, IGF1R, IGF2, IGFALS, IGSF1, IHH, IL1R1, INO80, IQCE, IRF6, IRS2, JAK,* ***JARID2****, K644M, KAT6B, KATNB1,* ***KDM4B****, KNG1, LARP7, LEP, LEPR, LHX3, LHX4, LIMBIN, LMNA, LOXL1, LTA, LTBP3, MC4R, MCM9, MEF2C , MLH, MOPD,* ***MOSPD1****, MRAP2, NDUFB3, NF1, NKX2, NMDA, NOS1AP, NPPC, NPR2, NPR3, NPRB, NR3C1, OBSL1, OTX1, PAPPA2, PAX8, PCNT, PIK3R1, PIT1, PLK4, PNKP, POC1A, POLD1, POMC, POP1, POU, POU1F1, PPP1R15B, PPP1R3A, PREDICT, PRKG2, PRLHR, PROP1, PSMD14, PTEN, PTEN, PTHLH, PTHRP, PTH,* ***PTPN11****, PYCR1, RAB33B, RAD26, RASA1, RDH16, RICTOR, RIOX1, RMRP, RNU4ATAC, ROR2, RSPRY1, RTTN, SBBYSS , SEMA3A, SEMD, SHOT, SHOX, SHOX, SIM1, SLC26A2, SLC29A3, SLC39A8, SMG1,* ***SMPD3****, SOCS1, SOCS2, SOCS3, SOX9, SQSTM1, STAT, STAT3, STAT5B, STC2, T354P, TALS, TANK, TBR1, TFIIH, THOX2, THRA, TITF1, TPO, TRH, TRHR, TRIM37,* ***TRPV4****, TSHR, TSP5, TSSK5, TTF1, TXNDC2, UBR1, VDR, WNT5A, XIST, XPBC, XPD, XPG, XRCC4, XYLT1, ZFP14, ZHX3*

*The genes involving ASSVs are marked in bold.

Supplementary Table 20. Quality control and statistics for Hi-C data.

**NGS-Filtering**	Read Length (bp)	100
	Raw Paired-end Reads	947,019,703
	Clean Paired-end Reads	945,469,044
	Clean Bases(bp)	189,093,808,800
	Clean Paired-end Reads Rate (%)	99.80%
	Clean Q30 Bases Rate (%)	95%
**Mapping**	Clean Paired-end Reads	945,469,044
	Unmapped Paired-end Reads	7,770,861
	Unmapped Paired-end Reads Rate (%)	0.82
	Paired-end Reads with Singleton	68,611,114
	Paired-end Reads with Singleton Rate (%)	7.3
	Multi Mapped Paired-end Reads	244,929,618
	Multi Mapped Ratio (%)	25.91
	Unique Mapped Paired-end Reads	624,157,451
	Unique Mapped Ratio (%)	66.02
**Hi-C filtering**	Unique Mapped Paired-end Reads	624,157,451
	Dangling End Paired-end Reads	13,005,404
	Dangling End Rate (%)	2.08
	Self Circle Paired-end Reads	1,095,487
	Self Circle Rate (%)	0.18
	Dumped Paired-end Reads	29,126,839
	Dumped Rate (%)	4.67
	Valid Paired-end Reads	579,564,619
	Valid Rate (%)	92.86%

Supplementary Table 21. Arguments and command lines of variants calling and filtering for WGS data.

<table>
<tr><td rowspan="9">Variant Calling</td><td colspan="2">Algorithm</td><td>Version</td><td>Command line</td></tr>
<tr><td colspan="2">BWA-MEM</td><td>0.7.12</td><td>bwa mem ref.fa read1.fq read2.fq</td></tr>
<tr><td rowspan="3">SNVs</td><td rowspan="2">GATK</td><td rowspan="2">3.6</td><td>java -jar GenomeAnalysisTK.jar -T</td></tr>
<tr><td>-I dedup.bam --emitRefConfidence GVCF -o</td></tr>
<tr><td>Samtools</td><td>1.3.1</td><td>Samtools mpileup -go 10macaque.by120.bcf -uf</td></tr>
<tr><td rowspan="4">SVs</td><td rowspan="4">Delly</td><td rowspan="4">0.7.7</td><td>delly call -t DEL -ref.fa dedup.bam -o DEL.bcf</td></tr>
<tr><td>delly call -t DUP -ref.fa dedup.bam -o DUP.bcf</td></tr>
<tr><td>delly call -t INS -ref.fa dedup.bam -o INS.bcf</td></tr>
<tr><td>delly call -t INV -ref.fa dedup.bam -o INV.bcf</td></tr>
<tr><td rowspan="17">Variant Filtering</td><td colspan="2">Tools</td><td>Site Filtering</td><td>Genotype Filtering</td></tr>
<tr><td rowspan="8">SNPs</td><td rowspan="7">GATK</td><td>java -jar GenomeAnalysisTK.jar \</td><td rowspan="7">python genotype_filter.py "DP:15-100"
"GQ>30" VariantCalls.filtered.vcf.gz</td></tr>
<tr><td>-T VariantFiltration \</td></tr>
<tr><td>-R ref.fa \</td></tr>
<tr><td>-V VariantCalls.SNP.vcf \</td></tr>
<tr><td>--filterExpression "QD < 2.0 || FS > 60.0 || MQ < 30.0 || MQRankSum < -12.5 || ReadPosRankSum < -8.0 " \</td></tr>
<tr><td>--filterName "my_snp_filter" \</td></tr>
<tr><td>-o VariantCalls.filtered.SNP.vcf</td></tr>
<tr><td>Samtools</td><td>vcffilter -f "MQ >30 & RPB >0.001 & MQB >0.001 & BQB >0.001 & MQSB >0.001" VariantCalls.SNP.vcf.gz</td><td>-</td></tr>
<tr><td rowspan="8">INDELs</td><td rowspan="7">GATK</td><td>java -jar GenomeAnalysisTK.jar \</td><td rowspan="7">python genotype_filter.py "GQ>30"
VariantCalls.filtered.vcf.gz</td></tr>
<tr><td>-T VariantFiltration \</td></tr>
<tr><td>-R ref.fa \</td></tr>
<tr><td>-V VariantCalls_chr$i.INDELS.vcf \</td></tr>
<tr><td>--filterExpression "QD < 2.0 || FS > 200.0 || ReadPosRankSum < -20.0" \</td></tr>
<tr><td>--filterName "my_indel_filter" \</td></tr>
<tr><td>-o VariantCalls.filtered.INDELs.vcf</td></tr>
<tr><td>Samtools</td><td>vcffilter -f "MQ >30 & MQSB >0.001" VariantCalls_chr$i.INDELS.vcf.gz | bgzip > hardfilter.INDELS.vcf.gz</td><td>-</td></tr>
<tr><td></td><td>SVs</td><td>Delly</td><td>QUAL >=20; SR >0; DP>3; marked PASS and PRECISE tag;</td><td>-</td></tr>
</table>

Supplementary Table 22. ICE clustering.

Term	Value
Total number of insert reads	5,232,764
Total number of 3'-primer reads	3,263,628
Total number of 5'-primer reads	3,401,423
Total number of poly-A reads	3,213,332
Total number of filtered short reads	1,438
Total number of non-full-length reads	2,607,116
Total number of full-length reads	2,624,210
Total number of full-length non-chimeric (FLNC) reads	2,468,473
Total base of number of full-length non-chimeric reads (bp)	6,809,351,534
Mean length of full-length non-chimeric read (bp)	2,759
Total number of raw consensus reads	1,275,860
Total bases of raw consensus reads (bp)	3,533,020,845
Total number of raw consensus reads	2,769
Total number of HQ>99% consensus reads	143,341
Total bases of HQ>99% consensus reads (bp)	396,323,333
Mean length of HQ>99% consensus reads (bp)	2,764
Total number of NGS_corrected reads	1,275,860
Total bases of NGS_corrected reads (bp)	3,561,028,798
Mean length of NGS_corrected reads (bp)	2,791
