# Supplementary material for: Long-read assembly of the Chinese rhesus macaque genome and identification of ape-specific structural variants
Source: Nat Commun. 2019 Sep 17;10:4233. doi: 10.1038/s41467-019-12174-w (PMC6749001; doi:10.1038/s41467-019-12174-w)
Supplement: Supplementary file 3 — Reporting Summary [file 41467_2019_12174_MOESM3_ESM.pdf]

nature research

Corresponding author(s): Bing Su

Last updated by author(s): Aug 19, 2019

Reporting Summary

Nature Research wishes to improve the reproducibility of the work that we publish. This form provides structure for consistency and transparency in reporting. For further information on Nature Research policies, see Authors & Referees and the Editorial Policy Checklist.

Statistics

For all statistical analyses, confirm that the following items are present in the figure legend, table legend, main text, or Methods section.

n/a	Confirmed	
☐	☒	The exact sample size (*n*) for each experimental group/condition, given as a discrete number and unit of measurement
☐	☒	A statement on whether measurements were taken from distinct samples or whether the same sample was measured repeatedly
☐	☒	The statistical test(s) used AND whether they are one- or two-sided<br>*Only common tests should be described solely by name; describe more complex techniques in the Methods section.*
☐	☒	A description of all covariates tested
☐	☒	A description of any assumptions or corrections, such as tests of normality and adjustment for multiple comparisons
☐	☒	A full description of the statistical parameters including central tendency (e.g. means) or other basic estimates (e.g. regression coefficient) AND variation (e.g. standard deviation) or associated estimates of uncertainty (e.g. confidence intervals)
☐	☒	For null hypothesis testing, the test statistic (e.g. *F*, *t*, *r*) with confidence intervals, effect sizes, degrees of freedom and *P* value noted<br>*Give P values as exact values whenever suitable.*
☒	☐	For Bayesian analysis, information on the choice of priors and Markov chain Monte Carlo settings
☒	☐	For hierarchical and complex designs, identification of the appropriate level for tests and full reporting of outcomes
☐	☒	Estimates of effect sizes (e.g. Cohen's *d*, Pearson's *r*), indicating how they were calculated

Our web collection on statistics for biologists contains articles on many of the points above.

Software and code

Policy information about availability of computer code

Data collection	We generated multi-plaform sequencing data of a male Chinese rhesus, involving tools including PacBio: Sequel (https://www.pacb.com/products-and-services/sequel-system/); Bionano: saphyr (https://bionanogenomics.com/products/saphyr/); Hi-C: Illumina X10; Iso-seq: Sequel; RNA-seq: Illumina X10.
Data analysis	FALCON (v1.8.7): https://github.com/falconry/falcon<br>Solve (v3.1): https://bionanogenomics.com/support-page/bionano-solve<br>Arrow(v2.3.0):https://github.com/PacificBiosciences/GenomicConsensus<br>Jellyfish (v1.1.11): https://github.com/gmarcais/Jellyfish<br>HiC-Pro (v2.8.1): https://github.com/nservant/HiC-Pro<br>LACHESIS (v1.0): https://github.com/shendurelab/LACHESIS<br>MUMmer (v.4.0): https://github.com/mummer4/mummer<br>BWA (v0.7.12): http://bio-bwa.sourceforge.net/)<br>GATK (v3.6): https://software.broadinstitute.org/gatk/)<br>SAMtools (v1.3.1): http://samtools.sourceforge.net/)<br>FastQC (v0.11.6): https://github.com/s-andrews/FastQC<br>Delly (v0.7.7): https://github.com/dellytools/delly<br>MAKER (v2.31.8): http://www.yandell-lab.org/software/maker.html<br>Augustus (v3.2.2): https://github.com/opendatagroup/augustus<br>SNAP (v1.0): https://github.com/lcoghill/snap<br>BUSCO (v3.0.1): https://gitlab.com/ezlab/busco<br>tRNAscan-SE (v1.0): https://github.com/yookuda/docker_trnascan_se<br>RNAmmer (v1.2): http://www.cbs.dtu.dk/services/RNAmmer<br>LoRDEC (v1.0): https://github.com/kamimrcht/ELECTOR<br>GMAP (v1.0): https://github.com/juliangehring/GMAP-GSNAP<br>Bowtie2 (v2.3.4.3): https://github.com/BenLangmead/bowtie2

HISAT2 (v2.1.0): https://github.com/infphilo/hisat2/releases
Tandem Repeats Finder (TRF, v4.07b): http://tandem.bu.edu/trf/trf.html
RepeatMasker (v4.0.6): https://github.com/rmhubley/RepeatMasker
IGV (Integrative Genomics Viewer) http://software.broadinstitute.org/software/igv
LiftOver (https://genome.ucsc.edu/cgi-bin/hgLiftOver)
SVTyper (v0.7): https://github.com/hall-lab/svtyper
smartie-sv (v1.0): https://github.com/zeeev/smartie-sv
VEP (Variant Effect Predictor): http://asia.ensembl.org/info/docs/tools/vep/index.html
DAVID (v6.8): https://david.ncifcrf.gov
LASTZ (v1.04): https://github.com/lastz/lastz
MEGA-X (v10.0.5): https://www.megasoftware.net
Primer Premier5 (v5.0): https://primer-premier-5.software.informer.com/

For manuscripts utilizing custom algorithms or software that are central to the research but not yet described in published literature, software must be made available to editors/reviewers. We strongly encourage code deposition in a community repository (e.g. GitHub). See the Nature Research guidelines for submitting code & software for further information.

Data

Policy information about availability of data

All manuscripts must include a data availability statement. This statement should provide the following information, where applicable:

- Accession codes, unique identifiers, or web links for publicly available datasets
- A list of figures that have associated raw data
- A description of any restrictions on data availability

The PacBio sequence data, Illumina sequencing reads, rheMacS assembly and its annotation files have been deposited in NCBI and GSA (Genome Sequence Archive) under the project accession numbers of PRJNA514196 and PRJCA001197, respectively.

Field-specific reporting

Please select the one below that is the best fit for your research. If you are not sure, read the appropriate sections before making your selection.

☒ Life sciences ☐ Behavioural & social sciences ☐ Ecological, evolutionary & environmental sciences

For a reference copy of the document with all sections, see nature.com/documents/nr-reporting-summary-flat.pdf

Life sciences study design

All studies must disclose on these points even when the disclosure is negative.

Sample size	An adult Chinese rhesus macaque (Macaca mulatta) (male, five-year-old) and five Chinese rhesus macaques for variant genotyping.
Data exclusions	No data were excluded.
Replication	No replication is need in this study.
Randomization	The all macaques used in this study are chose randomly.
Blinding	The investigators were not blinding to group allocations.

Reporting for specific materials, systems and methods

We require information from authors about some types of materials, experimental systems and methods used in many studies. Here, indicate whether each material, system or method listed is relevant to your study. If you are not sure if a list item applies to your research, read the appropriate section before selecting a response.

Materials & experimental systems

n/a	Involved in the study	
☒	☐	Antibodies
☒	☐	Eukaryotic cell lines
☒	☐	Palaeontology
☐	☒	Animals and other organisms
☒	☐	Human research participants
☒	☐	Clinical data

Methods

n/a	Involved in the study	
☒	☐	ChIP-seq
☒	☐	Flow cytometry
☒	☐	MRI-based neuroimaging

Animals and other organisms

Policy information about studies involving animals; ARRIVE guidelines recommended for reporting animal research

Laboratory animals	An adult Chinese rhesus macaque (Macaca mulatta) (male, five-year-old) and five unrelated Chinese rhesus macaques.
Wild animals	*Provide details on animals observed in or captured in the field; report species, sex and age where possible. Describe how animals were caught and transported and what happened to captive animals after the study (if killed, explain why and describe method; if released, say where and when) OR state that the study did not involve wild animals.*
Field-collected samples	*For laboratory work with field-collected samples, describe all relevant parameters such as housing, maintenance, temperature, photoperiod and end-of-experiment protocol OR state that the study did not involve samples collected from the field.*
Ethics oversight	All animal protocols were approved in advance by the Institutional Animal Care and Use Committee of Kunming Institute of Zoology (Approval No: SYDW-2010002).

Note that full information on the approval of the study protocol must also be provided in the manuscript.
