# Supplementary material for: Long-read assembly of the Chinese rhesus macaque genome and identification of ape-specific structural variants
Source: Nat Commun. 2019 Sep 17;10:4233. doi: 10.1038/s41467-019-12174-w (PMC6749001; doi:10.1038/s41467-019-12174-w)
Supplement: Supplementary file 4 — Description of Additional Supplementary Files [file 41467_2019_12174_MOESM4_ESM.pdf]

Description of Additional Supplementary Files

Supplementary Data 1. RNA-seq data from 16 macaque tissues aligned to genome assemblies.

Supplementary Data 2. Comparison of repeat contents between rheMacS, rheMac8 and apes.

Supplementary Data 3. Summary of SVs in rheMacS.

Supplementary Data 4. Summary of ASSVs.

Supplementary Data 5. Functional annotation clustering analysis for ASSVs.

Supplementary Data 6. The 25 ASSVs located in coding regions.

Supplementary Data 7. Summary of the 7,155 ADEs in eight brain regions.

Supplementary Data 9. Summary of ASSVs located in ASP and GASP genes.

Supplementary Data 10. All primers used in this study.
